# The adjunct role of pharmacotherapy in multimodal treatment of paediatric functional neurological disorder

**DOI:** 10.3389/fpsyt.2025.1560873

**Published:** 2025-07-17

**Authors:** Rynagh Cummins, Clare Hawkes, Judy Longworth, Stephen Scher, Kasia Kozlowska

**Affiliations:** ^1^ Department of Psychological Medicine, The Children’s Hospital at Westmead, Sydney, NSW, Australia; ^2^ Center School of Psychological Sciences, University of Tasmania, Launceston, TAS, Australia; ^3^ Child Youth Mental Health Service North (CYMHSN), Launceston, TAS, Australia; ^4^ Department of Psychiatry, Harvard Medical School, and McLean Hospital, Belmont, MA, United States; ^5^ University of Sydney Medical School, Sydney, NSW, Australia; ^6^ The Children’s Hospital at Westmead, and Disciplines of Psychiatry and Child and Adolescent Health, University of Sydney Medical School, Sydney, NSW, Australia; ^7^ Brain Dynamics Centre, Westmead Institute for Medical Research, Westmead, NSW, Australia

**Keywords:** arousal, child, functional neurological (conversion) disorder (FND), medication, functional/dissociative seizures, sleep problems, psychiatry, rehabilitation

## Abstract

**Background:**

Rehabilitation for children with functional neurological disorder (FND) requires a biopsychosocial intervention: physiotherapy, psychotherapy, pharmacotherapy, school attendance, and family work. This study documents the pharmacotherapeutic element and its rationale.

**Methods:**

Medication use was documented in 158 children with FND (41 boys, 117 girls, aged 8.50–17.58; mean 13.78) admitted into the Mind-Body Program.

**Results:**

On presentation, children with FND had high levels of functional impairment, school loss, and comorbid psychiatric, functional, and medical disorders. On admission, 63% (n=95) were on medications. During admission, 130 (82.3%) children had pharmacotherapy interventions: dose adjustment, initiation, or discontinuation. 88.6% (n=140) were discharged on medications. Pharmacotherapy targets included: antidepressants for anxiety/depression (n=111; 70.3%); antipsychotics for extreme anxiety/arousal (n=73; 46.2%); melatonin for sleep (n=64; 40.5%); α agonists and β blockers, for arousal, sleep initiation, and trauma-related nightmares (n=58; 36.7%); iron/vitamin supplementation (n=30; 19.0%); and medications for functional gut symptoms (n=28; 17.7%) and comorbid pain (n=20; 12.7%).

**Conclusions:**

Pharmacotherapy is used as an adjunct in paediatric FND to down-regulate the stress system, reset the circadian clock, manage pain, and treat comorbid disorders. Pharmacotherapy and its concomitant placebo effects scaffold the child to enable engagement in all components of the therapeutic process and return to healthy function.

## 1 Introduction

Functional neurological disorder (FND) is a neuropsychiatric disorder that presents with a myriad of neurological symptoms that reflect aberrant changes within and between neuron-glial (brain) networks (1). In children (including adolescents), presentations are heterogeneous and involve multiple domains: FND symptoms and symptom combinations; comorbid functional somatic symptoms (e.g., pain, fatigue, orthostatic intolerance); comorbid anxiety, depression, and other mental health disorders; comorbid functional and medical disorders; and finally, predisposing, precipitating, and perpetuating factors, which include high rates of adverse childhood experiences (ACEs), psychological distress, and family stress (2, 3). At the more extreme end of the illness spectrum, children experience high levels of disability and are unable to go to school or to engage independently in skills of daily living. If resources allow, the child may be offered treatment in an inpatient rehabilitation program (2, 4). Such programs offer a multimodal treatment intervention made up of modules that target particular areas of dysfunction: physiotherapy, psychotherapy, pharmacotherapy, social skills–based group work, attendance/reintegration in school, and working with the family. The modules are delivered simultaneously or sequentially during the inpatient admission and subsequently in the outpatient setting. Using this approach, changes through therapy are achieved as a “function of combined techniques, strategies and modalities” (p. 11) (5). The current study examines the pharmacotherapeutic element of the rehabilitation intervention and its rationale in 158 children treated via the Mind-Body Program at The Children’s Hospital at Westmead, in Sydney, Australia.

**Figure 1:**
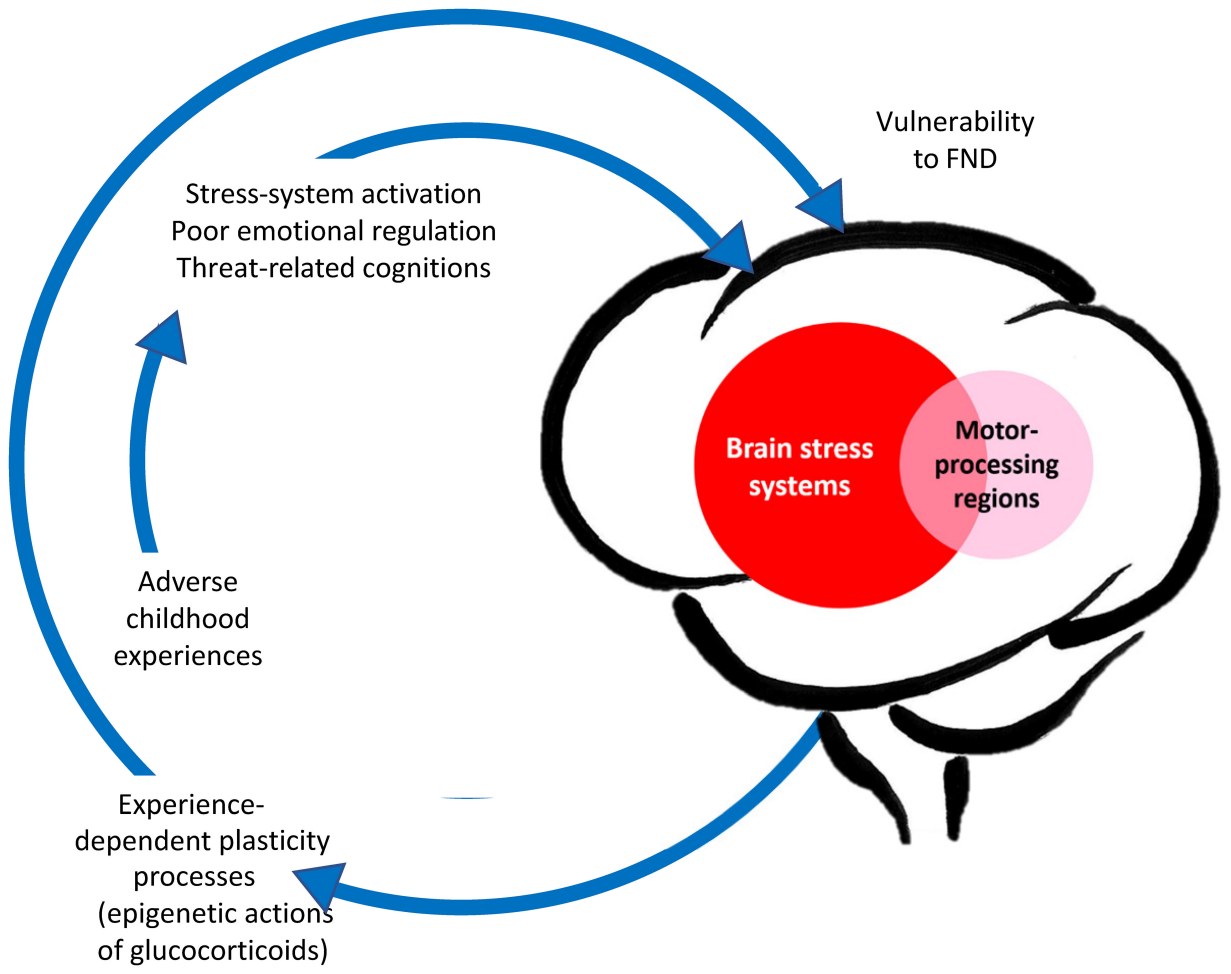
Aetiological model of paediatric FND: Visual representation linking adverse life experiences, stress system activation, and epigenetic/plasticity processes that increase vulnerability for FND. ^©^ Kasia Kozlowska 2021.

**Figure 2:**
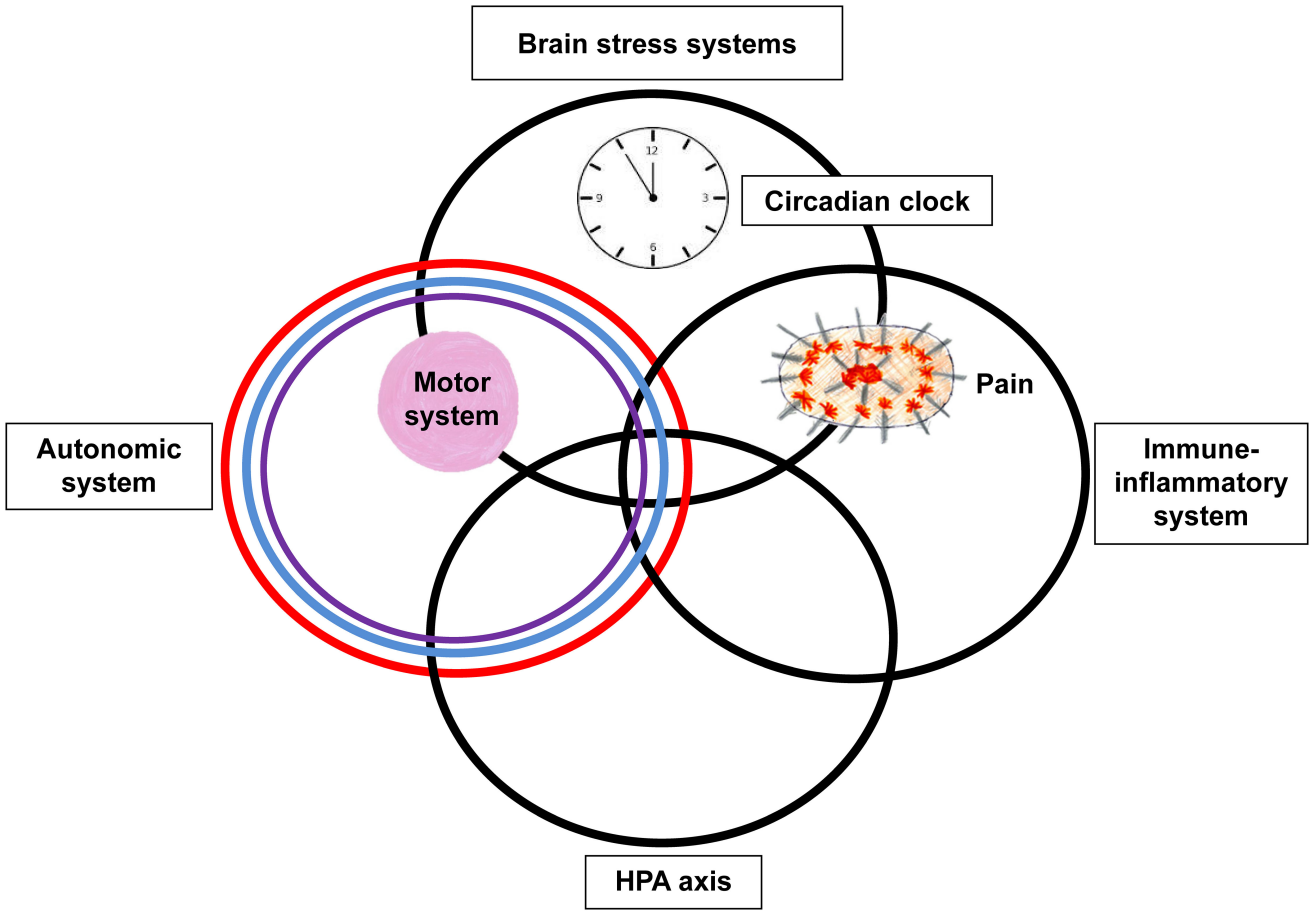
Circles metaphor of the stress-system model for functional somatic symptoms (including FND). This figure depicts the various components of the stress system that may be dysregulated in the child with FND and that may need to be targeted during the therapeutic intervention (including pharmacotherapy). The overlap between the different components of the stress system – the HPA axis, autonomic nervous system, immune-inflammatory system, and brain stress systems – is presented by the overlap between the circles. The circadian clock is placed within the top circle because the master clock is found in the hypothalamus, a small region located in the base of the brain. The motor system, which includes central and peripheral components, is represented by the pink ball. The placement of the pink ball in the overlap between the brain stress systems and autonomic system reflects that activation of these systems can be accompanied by changes in motor function. The pain system, which also includes central and peripheral components, is represented by the spiky oval. The placement of pain in the overlap between the brain stress systems and immune-inflammatory system reflects that activation of these systems maintains chronic complex pain. ^©^ Kasia Kozlowska 2013.

The neurobiology of paediatric FND is complex (3). The changes in neuron-glial networks that underpin FND are thought to be mediated by complex interactions between brain, mind, body, and context—the lived experience of the child and the family. Stress-system activation and epigenetic processes enable lived experience to be biologically embedded in the body and brain, resulting in dysregulation of neural networks and presentation with symptoms of FND (See **Figure 1**). Factors that activate or further dysregulate the child’s stress system—disrupted sleep, illness-promoting psychological processes, poor regulation capacities, low stress tolerance, physical deconditioning, anxiety, depression, and so on—are key drivers of the illness process. In the Mind-Body Program, clinicians use the stress-system model (6) to understand these various factors and to guide assessment, formulation, and treatment (see **Figure 2**). The model helps clinicians to identify specific areas of dysfunction and to deliver treatment modules that address those factors as they pertain to the individual, family, and school and social-system levels. Other contemporary systemic (biopsychosocial) models of FND include the stress-diathesis model (7, 8), the two-hit neuropathophysiological model (9), and the three-hit model of developmental trajectories of early life stress (3, 10). The common thread across all these models is that they provide a framework for considering the interactions between multiple factors—biological, psychological, and social.

While there are no known medications for the treatment of FND, pharmacotherapy can be a useful adjunct that helps to manage stress-system dysregulation or co-existing conditions or symptoms. In the following sections, we identify common problem areas that may potentially involve the adjunct use of medication in some children. The overarching rationale is that medication is used to support the therapeutic process and to address underlying “drivers” that contribute to stress-system activation and the maintenance of FND symptoms. Medication in FND is never a stand-alone intervention: it is used alongside other interventions—involving the child, family, and school—to support the treatment process. Below we outline some of the driving factors that contribute to the FND presentation, where adjunct medication can play a useful role.

### 1.1 Driver 1: disrupted sleep

A recurring theme raised by the children admitted to the Mind-Body Program is that of bad sleep: difficulties falling asleep, multiple night wakings, or sleep that is unrefreshing. As clinicians, we typically observe that after a night of bad sleep, the child’s pain is worse, the child feels more nauseous, the child’s fatigue is more pervasive, the child is more likely to have a functional seizure—if functional seizures are part of the presentation—and the child feels more *off* and *ikky* in general.

In a study looking at sleep—which included 32 children from the current cohort as participants—we found that children with FND (*vs*. controls) had an attenuated cortisol awakening response (CAR) or an obliterated/reversed CAR (11). CAR was negatively correlated with ACEs and subjective distress. These findings suggest that in the context of cumulative stress, children with FND suffer from dysregulation of the circadian clock, resulting in poor sleep and thereby compromising the myriad restorative functions of sleep. More broadly, since every organ, tissue, and cell in the body, as well as every component of the stress system, has a circadian rhythm, a dysregulated circadian clock compromises the function of every body system. In our Mind-Body Program we take the circadian clock to be a *nodal point* for intervention—that is, an intervention “that result[s] in a greater degree of change than others or are so fundamental that without [it], the desired progress will not be achieved” (p.285) (6). If FND reflects a neurophysiological state of dysregulation, then regulating the circadian clock is the first step in the therapeutic process of trying to regain physiological coherence (12) across body systems. Consequently, sleep interventions—sleep hygiene strategies supported by pharmacotherapy when needed—are a crucial initial target of the Mind-Body Program, our FND rehabilitation program.

### 1.2 Driver 2: mental health issues

The rates of comorbid mental health disorders in children with FND vary substantially from cohort to cohort (22%–80%) (2). Because the Mind-Body Program serves children who are functionally impaired—the more severe end of the illness spectrum—comorbid mental health disorders are common. And in cases where these comorbid disorders fail to resolve with treatment, they have a detrimental effect on outcomes (13).

Mental health disorders may complicate the child’s FND presentation in various ways. Illness-promoting psychological processes and attention to illness cues—more likely to be present in children with anxiety, autism spectrum disorder (ASD), depression, and posttraumatic stress disorder (PTSD)—activate the stress system in a top-down fashion (14–16). Difficulties with motivation, energy, anhedonia, and self-harm, as seen in major depression, can hamper rehabilitation efforts. States of high arousal seen in children with ASD contribute to stress-system activation and be difficult to downregulate (17, 18). Unresolved symptoms of trauma—nightmares, flashbacks, and hypervigilance symptoms—may keep the child in a state of constant fear and arousal. Given these findings, the treatment of psychiatric symptoms or disorders is an important element of any paediatric FND rehabilitation program.

### 1.3 Driver 3: comorbid functional symptoms and syndromes

Comorbid functional symptoms and syndromes—many of which involve stress-system activation—can complicate the FND presentation through a variety of pathways. Comorbid complex/chronic pain or chronic functional gut symptoms (e.g., constipation with abdominal pain or persistent nausea) affect function and efforts at rehabilitation. Comorbid functional somatic symptoms have a vortex-like effect: they capture attention, magnify anxiety, and amplify the whole constellation of symptoms (including those of FND). Consequently, interventions that help contain comorbid functional symptoms (e.g., manage comorbid constipation or dampening down persistent nausea) may be important targets of any paediatric FND rehabilitation program.

## 2 Methods

### 2.1 Participants

One hundred and fifty-eight children received treatment for FND via the Mind-Body Program at The Children’s Hospital at Westmead, Sydney, Australia, during the period September 2006 to September 2024. A typical Mind-Body Program admission—run from a paediatric medical ward of the hospital—runs over a two-week period. All children had previously undergone a comprehensive neurology assessment and had been diagnosed with FND by a paediatric neurologist using criteria from the *Diagnostic and Statistical Manual of Mental Disorders* (DSM). DSM-IV-TR diagnoses were used for the 2006–18 study period (cohort 1; n = 64), and DSM-5 diagnoses for the 2018–24 study period (cohort 2; n = 94) (19, 20). Participants included in the study also agreed to participate in the FND research program.

All participants with FND (and their families) took part a biopsychosocial assessment with the mind-body team. The assessment involves a semi-structured interview with the child and family documenting the following: the child’s developmental history; history of the presenting symptoms (including comorbid nonspecific symptoms, quality of sleep, functional syndromes, and mental health concerns); school attendance/loss; quality of sleep; medications used by the child; and child’s level of functional disability (on the Global Assessment of Functioning [GAF] scale) (see **Table 1**). The mental state assessment begun during the structured interview was continued into the admission process, yielding comorbid mental health diagnoses for a subset of patients. Resting-state heart rate (HR) and respiratory rate (RR) were also documented. For cohort 1 these rates were measured during a laboratory-based assessment (21), and for cohort 2, as part of the medical admission process. For children who complained of dizziness as part of their clinical presentation, a formal standing test was completed to rule in or rule out the diagnosis of comorbid postural orthostatic tachycardia syndrome (POTS) (22). A diagnosis of POTS—a manifestation of autonomic system dysregulation—was given if HR on ten minutes of standing from the supine position increased ≥40 beats per minute (with a blood pressure that remained stable). Because unhealthy eating patterns and issues with weight had been noted in the first cohort, weight centiles were collected in the second cohort.

**Table 1:** Summary of the measures used in the study.

Measure	Description
RAHC- GAF	The Royal Alexandra Hospital for Children Global Assessment of Function (RAHC-GAF) is the DSM-IV-TR GAF modified to include functional impairment secondary to physical illness (19). The scale has 100 points and 10 categories (10 points each). Healthy controls generally fall into the upper three brackets “superior in all areas” (score 91−100), or “good in all areas” (score 81−90). Lower values (and brackets) mark functional impairment of increasing severity. Patients with physical or psychological impairment fall into the lower brackets (score <81).
DASS-21	The Depression Anxiety and Stress Scales (DASS-21)—total DASS score, but not the three subscales—are a validated measure of perceived distress in paediatric populations (74, 75).
ELSQ	The Early Life Stress Questionnaire (ELSQ) is a checklist of 19 stress items—and an option for elaboration—based on the Child Abuse and Trauma Scale (76). Twelve items pertain to relational stressors, including the following: bullying; physical abuse; sexual abuse; emotional abuse; neglect; parental separation; loss by separation; loss by death; family conflict; severe illness of a family member; domestic violence; and other. Other items pertain to birth complications, life-threatening/severe illness, war trauma, and natural disasters. Participants record if they have or have not experienced the given stressor and the age period during which the stressor has been experienced.

On self-report the children completed the Depression Anxiety and Stress Scales (DASS-21) and Early Life Stress Questionnaire (ELSQ) (see **Table 1**).

Forty-seven healthy controls of a similar age and sex were recruited from the same geographical catchment area. Control participants were screened for the absence of mental health disorders, history of head injury, family history of mental health disorders, and chronic health concerns. All controls completed self-report measures—the DASS-21 and the ELSQ—and were rated on the GAF. The control group was included to enable the data from the DASS and ELSQ—which do not have available norms for children—to be compared to control-group data.

Subgroups of participants with FND took part in parallel studies examining a range of biomarkers in an effort to better understand the neurobiology of FND (3). These studies examined the following: electrocardiogram measures (markers of autonomic system arousal) (21); percutaneous carbon dioxide (pCO2) (marker of CO2 regulation following a hyperventilation challenge) (23); blood C-reactive protein (CRP) levels (marker of inflammation) (24); qualitative electroencephalogram (EEG) measures (marker of cortical arousal) (25, 26); CAR (marker of hypothalamic-pituitary-adrenal [HPA] axis function) (11); and brain-imaging studies (to examine changes in structure, neural-glial network function, and neurometabolite concentrations and dependencies) (1, 27–29).

The Royal Alexandra Hospital for Children (later The Children’s Hospital at Westmead) Human Research Ethics Committee and the Sydney Children’s Hospital Network Human Research Ethics Committee approved the first and second study, respectively. Participants and their legal guardians provided written informed consent.

### 2.2 Analysis of clinical characteristics and self-report data

Chi-square analyses and independent t-tests were used to calculate differences between the FND and control groups on categorical and continuous variables, respectively.

## 3 Results

### 3.1 Missing data

In the FND group, missing data included: HR (n = 3), RR (n = 20), weight centile (n = 64), DASS-21 (n = 19), and ELSQ (n = 19). HR and RR were not collected in healthy controls. HR and RR centiles were established using published centile charts (30).

Paracetamol was excluded from all analyses because, if used, it was used pro re nata (PRN), which was not well reported or documented for paracetamol. Amitriptyline was excluded from the analyses about antidepressants because, in the four children who were prescribed amitriptyline on admission, it had previously been used in low doses for the indication of chronic complex pain and not depression.

### 3.2 The clinical characteristics of participants with FND

**Figure 3:**
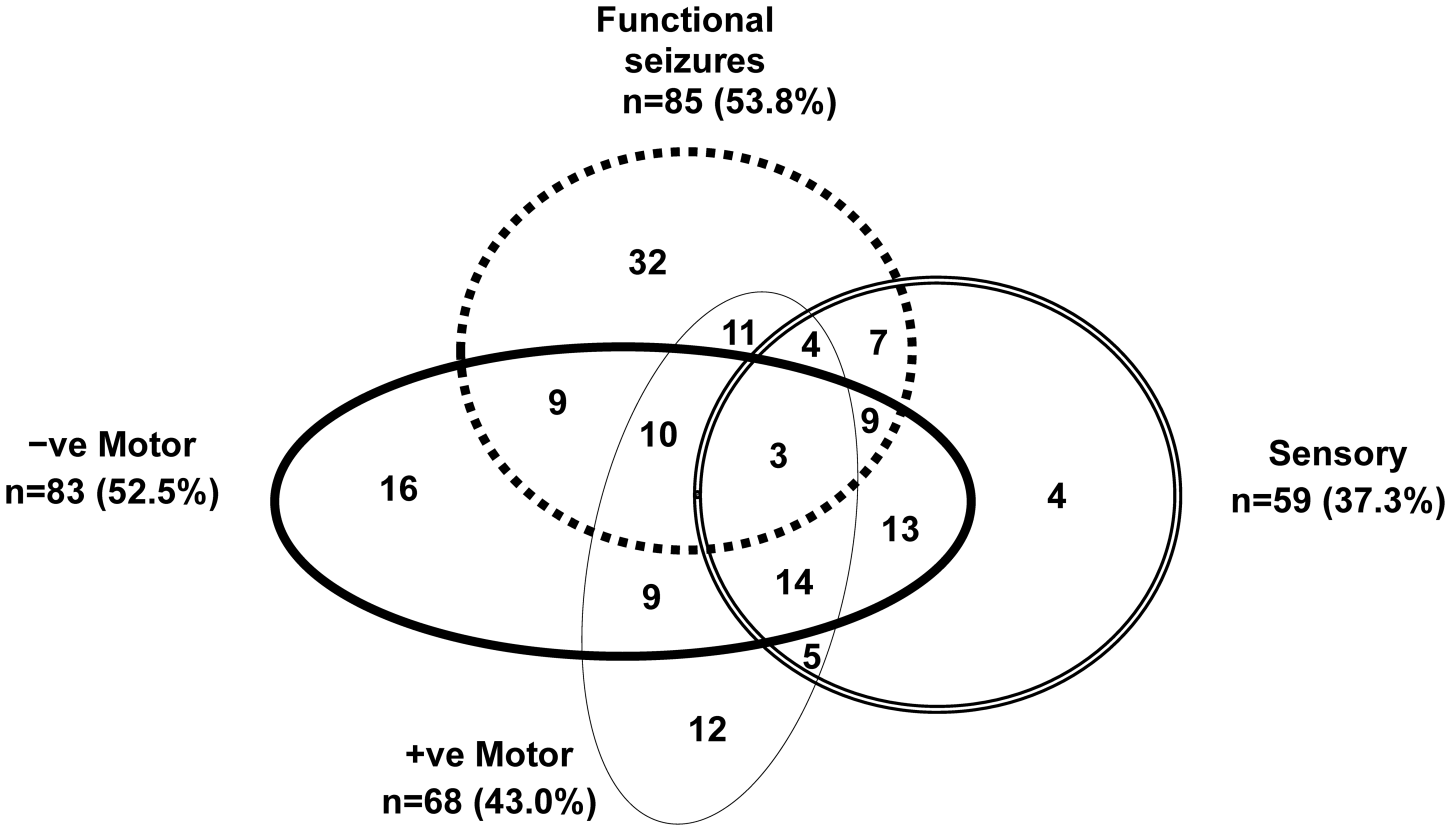
Visual representation of functional neurological symptoms experienced by the children in the study cohort. Children with mixed FND commonly present with multiple functional neurological symptoms. This figure depicts the functional neurological symptoms experienced by the 158 children with FND who were included in the analysis. Negative motor symptoms included: weakness or loss of function in the limbs, aphonia (loss of voice), and difficulties swallowing. Positive motor symptoms included: unusual gaits, difficulties with balance coupled with an uncoordinated gait, tics, tremors, dystonia, rumination (bringing up food via overactivation of the diaphragm), and dysphonia (change in the quality of the voice, e.g., a high-pitched baby voice). Sensory symptoms included: loss of touch, hearing, or vision. Functional seizures presented in a broad variety of ways and included faint-like events.

The clinical presentations of the participants with FND (n = 158) were diverse. The children presented with one or more functional neurological symptoms (range, 1–8; mean = 2.66; median = 2.00) (**Figure 3**). Length of illness ranged from 2 days to 4 years (mean = 5.49 months; median = 4.00 months): 70.9% had been ill for less than six months. With the exception of outliers, length of admission was generally two weeks (median 2 weeks, mean 2.6 weeks, range 1–18 weeks). Levels of functional disability were high, with GAF scores—100 being the best possible score—ranging from 10 to 65 (mean = 35.39; median = 35). Weeks of school loss ranged from 0 to 52 (mean = 8.46; median = 4.00). The premorbid functioning of the majority of participants fell within the normal IQ range (see **Table 2**).

**Table 2:** Clinical and demographic information about participants with FND from clinical assessment.

Comorbid medical conditions		
Any comorbid medical condition	29	18.35%
- Asthma/allergies	15	9.5%
- Epilepsy	9	5.7%
- Complex regional pain syndrome (CRPS)	8	5.1%
- Hypermobility	4	2.5%
- Tourette’s syndrome	4	2.7%
- Migraine	3	1.9%
- Benign intracranial hypertension	2	1.3%
- Hashimoto’s disease	2	1.3%
- Kidney disease	2	1.3%
- Cerebral palsy	1	0.6%
- Neurofibromatosis (NF1)	1	0.6%
- Transverse myelitis (triggering FND symptoms alongside)	1	0.6%
- Narcolepsy	1	0.6%
- Congenital heart disease	1	0.6%
- Congenital cataract	1	0.6%
- Celiac disease	1	0.6%
Comorbid functional syndromes		
Complex (functional) pain	105	66.5%
- Headache	54	34.2%
- Lower limbs	34	21.5%
- Abdomen	20	12.7%
- Back/neck	18	11.4%
Any diagnosed comorbid functional syndrome	53	33.6%
- Functional gut disorder	37	23.4%
- Postural orthostatic tachycardia syndrome (POTS)*	24	15.2%
- Irritable bladder	1	0.6%
Comorbid non-specific somatic symptoms		
Any comorbid non-specific somatic symptom	123	77.8%
Fatigue	81	51.3%
Dizziness	76	48.1%
Nausea	55	34.8%
Breathlessness	42	26.6%
Comorbid difficulties with sleep		
Difficulties with sleep reported by the child	116	73.4%
Comorbid mental health disorders		
Any mental-health/neurodevelopmental disorder (DSM-5)	135	85.4%
- Anxiety disorder	110	69.6%
- Depressive disorder	55	34.8%
- Autism	19	12.0%
- PTSD	18	11.8%
- Learning difficulties	12	7.6%
- Dissociative disorder	8	5.1%
- ADHD	13	8.2%
- Oppositional defiant disorder	5	3.2%
- Gender dysphoria	3	1.9%
- Eating disorder	2	1.3%
- Bipolar disorder	2	1.3%
- Psychosis (first episode of psychosis)	2	1.3%
- Skin Picking disorder	2	1.3%
- Conduct disorder	1	0.6%
- Enuresis	1	0.6%
Common adverse childhood experiences (ACEs) reported by the child and family		
One or more ACEs (range 1–12, mean 5.63, median 6)	158	100%
- Family conflict	92	58.2%
- Bullying by peers	92	58.2%
- Child physical illness	81	51.3%
- Maternal mental illness	70	44.3%
- Loss via separation from a loved one or a close friend	63	39.9%
- Loss via death of a loved one	49	31.0%
- Paternal mental illness	41	25.9%
- Exposure to domestic violence	37	23.4%
- Maternal physical illness	37	23.4%
- Frequent moves of house	35	22.2
- Paternal physical illness	29	18.4%
- Exposure to domestic violence	6	19.4%
- Emotional abuse (e.g., rejection/abandonment by a parent)	19	12.0%
- Physical abuse	17	10.8%
- Neglect	15	9.5%
- Sexual abuse	13	8.2%
- Custody battle	11	7.0%
- Migration	6	3.8%
Intelligence quotient (IQ)**		
- Superior	39	24.7%
- Average	105	66.5%
- Borderline	11	7.0%
- Delayed	3	1.9%

*POTS in our population is placed under functional disorders because it is secondary to dysregulation of the autonomic system and not due to an organic pathology (e.g., neuropathy).

**IQ had been formally tested in 50 children (31.6%); was estimated from the Spot-the-word Test completed during the IntegNeuro Battery of cognitive tests in 56 children (35.4%); and was estimated from a formal educational assessment at the Hospital School, previous school reports, and formal Department of Education testing of literacy and numeracy, in 52 children (32.9%).

Comorbid functional disorders were present in one-third of children (n = 53; 33.6%), with functional gut disorders (n = 37; 23.4%) being the most common (see **Table 2**) (31). Comorbid pain was present in two-thirds (n = 105; 66.5%). Other comorbid, nonspecific functional symptoms—fatigue, dizziness, nausea, breathlessness—were also common (n = 123; 77.8%) (see **Table 2**). On assessment, almost two-thirds of children (n = 99; 63.7%) reported difficulties with sleep initiation or maintenance.

Comorbid medical conditions were present in 49 children (31.0%; see **Table 2**). Most common disorders were asthma (n = 15; 9.5%), epilepsy (n = 9; 5.7%), and chronic regional pain syndrome (CRPS) (n = 8; 5.1%). Eight of the children with epilepsy presented with functional seizures as part of their FND presentations. Four of the children with CRPS reported pain only in the effected lower limb; 3 reported pain in multiple regions (including the effected lower limb); and one reported that the pain in the effected leg had recently resolved. Comorbid mental health/neurodevelopmental disorders were identified (and treated) for 134 (84.8%) of children (range, 0–6; mean = 1.60; median = 1.00) (see **Table 2**). The most common comorbid diagnoses were anxiety (n = 110; 69.6%) and depression (n = 55; 34.8%). In one-fifth of children (n = 34; 21.5%), safety plans for managing suicidal ideation and self-harm behaviours were an important element of the clinical intervention.

Children with FND came from a range of family situations: intact families (n = 96; 60.8%), separated parents with the child living with the biological mother (n = 44; 27.8%), separated parents with the child living with the biological father (n = 15; 9.5%), and adoption/foster care (n = 3; 1.9%). Families spanned all socioeconomic classes: professional (n = 60; 38.0%), white collar (n = 47; 29.7%), blue collar (n = 43; 27.2%), and unemployed/on welfare (n = 8; 5.1%).

All families reported stressors across the child’s lifespan (range, 1–12; mean = 5.63; median 6.00) (see **Table 2**). Family conflict (n = 92; 58.2%), bullying by peers (n = 92; 58.2%), and child physical illness (n = 81; 51.3%) were the most common. One-fifth (n = 36; 22.8%) had experienced some form of maltreatment (physical abuse, sexual abuse, or neglect) during their lifespan (see **Table 2**). In 66 children (41.8%), a physical stressor was reported as a trigger event to the FND illness. Physical stressors included the following: an illness event (n = 30; 19% [14 of 30 being a viral illness]); injury of some sort (n = 25; 15.8% [4 of 25 being head injuries and 4 involving a fracture]); or a medical procedure (14; 8.9% [6 of 14 being a vaccination]). On the ELSQ, children with FND (*vs*. healthy controls) had higher scores on the DASS-21 (a measure of distress) and reported more adverse life events across development (see **Table 3**).

**Table 3:** Comparisons between FND and healthy-control groups on age, sex, global assessment of function (GAF), respiratory rate, heart rate, weight percentile, depression anxiety and stress scales (DASS-21), and early life stress questionnaire (ELSQ).

Measure	FND group (n=158) mean value/total score (range)	Healthy-control group (n=47) mean value/total score (range)	t/χ^2^ (p)
Age	13.78 8.50–17.58	13.84 8.58–17.92	-.159 (.874)
Sex	117 girls 41 boys	35 girls 12 boys	.003 (.954)
GAF	35.39 (median 35) (10–65)	89.60 (median 90) (75–99)	-32.01 (<.001)
RR (breaths/min) < 12 years (n = 26) ≥ 12 years (n = 112)	23.09 (median 20.00) (14.24-40.00) 19.92 (median 18.00) (9.00-50.00)	––	
HR (beats/minute) < 12 years (n = 32) ≥ 12 years (n = 123)	84.41 (median 86.30) (64-125) 85.02 (median 85.00) (60-118)	––	
Weight percentile	66.72 (4.48–100.00)	64.23 (11.00–99.15)	.519 (.604)
DASS-21 Score (n = 139)	20.98 (median 20) (0–52)	5.68 (median 4) 0–30	10.714 (<.001)
ELSQ (19 items) (n = 139)	4.22 (median 4) (0–16)	0.51 (median 0) (0–3)	12.31 (<.001)

RR on admission/laboratory assessment was documented in 138 children with FND (see **Table 3**). Based on published centile charts (30), our patients showed a shift to the right of the normative curve, with 45.65% (n = 63/138) ≥75th centile; 30.43% (n = 42/138) ≥90th centile; and 16.67% >100th centile. Those >100th centile had respiratory rates of 24–50 breaths per minute—that is, they were hyperventilating at the time of assessment.

HR on admission/laboratory assessment was documented in 155 children with FND (see **Table 3**). Based on published centile charts (30), 4 patients (2.6%) had HRs >100th percentile, and the rest 151 (97.4%) had HRs between the 5th and 97th percentiles.

For the 76 children (48.1%) who complained of dizziness, a 10-minute standing test (22, 32) was completed to identify the subgroup with POTS, a manifestation of autonomic system dysregulation marked by too much sympathetic activation and too little restorative vagal activation. The standing test uses standing from a lying position as “a stressor” to examine the response and regulation capacity of the autonomic nervous system. HR increases ≥40 beats per minute were documented in 24 children, consistent with a clinical diagnosis of POTS. Clinical HR measures—as documented routinely on the clinical observation chart of vital signs (done on admission and one daily in our patient group)—were insufficient for the task of identifying this group of children with POTS (HR centiles, 20–93; mean = 54.87; median = 55.00).

For a subgroup of 57 study participants (*vs*. age- and sex-matched healthy controls), a closer laboratory-based examination of electrocardiogram (ECG) data had documented a state of autonomic system activation at rest and decreased capacity for a healthy autonomic response in response to stressors. Compared to controls, patients had elevated HRs (reflecting increased sympathetic activation) and decreased heart rate variability (reflecting decreased vagal tone), and they were unable to mount an appropriate response (HR increase) when presented with a stressor (auditory oddball and Go/No-Go tasks). Formal resting-state skin conductance performed in this same group (*vs*. sex- and age-matched controls) showed increased skin conductance, which is also a measure of sympathetic activation (see Appendix in Savage et al., 2022) (33).

In sum, what our mind-body team learned from the above-described parallel study was that routine daily hospital documentation of vital signs—HR and blood pressure, coupled with the use of published centile charts—was insufficient to identify the autonomic arousal and the impaired capacity for autonomic regulation in our patients with FND. Consequently, our decisions concerning potential interventions (including medication) pertaining to the autonomic nervous system needed to be based on the presence (or not) of clinical symptoms signalling autonomic system dysregulation (6) or, in the case of POTS, the results of the standing test.

### 3.3 Medication use on admission to the Mind-Body Program

On admission to the Mind-Body Program, three-fifths of children (n = 95; 60.13%; mean = 1.70; median = 1.00) were taking medication prescribed by their family doctor, paediatrician, or paediatric neurologist (see **Table 4**). The most common medication groups were as follows: antidepressants for anxiety or depression (n = 55; 38.8%); atypical antipsychotic medication for extreme anxiety or arousal (including difficulties falling asleep) (n = 35; 22.2%); melatonin hormone supplement for sleep (n = 30; 19.0%); α agonists, β blockers, and hyperpolarization-activated, cyclic, nucleotide-gated (HCN) channel blockers for down-regulating arousal, helping with sleep initiation, or managing trauma-related nightmares or extreme HR increases in POTS (n = 25; 18.8%); medications for managing functional gut symptoms (n = 18; 11.4%); and medications for managing comorbid pain (n = 18; 11.4%).

**Table 4:** Medications on admission to the mind-body program.

Medication	FND cohort (n=158) on admission	FND cohort (n=158) on discharge	Rationale for use/dis-use at discharge
Supplements for management of the sleep-wake cycle			
Melatonin	30 (19.0%)	64 (40.5%)	Re-regulate circadian clock/sleep
α antagonists, α agonists, β blockers, and HCN channel blockers			
Any arousal-decreasing medication	25 (18.8%)	58 (36.7%)	
Clonidine α-2 agonist	13 (8.2%)	34 (21.5%)	Down-regulate arousal at the CNS level
Guanfacine α-2a agonist	8 (5.1%)	18 (11.4%)	Down-regulate arousal at the CNS level (n=14) ADHD symptoms (n=1) Both indications (n=3)
Prazosin Competitive α -1 antagonist	1 (0.6%)	3 (1.9%)	Decrease frequency of trauma-related nightmares & down-regulate arousal at the CNS level
Propranolol β blocker	6 (3.8%)	13 (8.2%)	Prevent steep HR elevations in children with POTS
Ivabradine HCN channel blocker	0	1 (0.6%)	Prevent steep HR elevations in children with POTS (without BP changes)
Antidepressants			
Any antidepressant	55 (34.8%)	111 (70.3%)	Management of anxiety or depression
Fluoxetine (SSRI)	34 (21.5%)	51 (32.3%)	Ease depression and anxiety symptoms; down-regulate autonomic (sympathetic) arousal; promote neuroplasticity; modulate inflammation and immune activation
Fluvoxamine (SSRI)	10 (6.3%)	36 (22.8%)	Ease depression and anxiety symptoms; down-regulate autonomic (sympathetic) arousal; promote neuroplasticity; modulate inflammation and immune activation
Sertraline (SSRI)	2 (1.3%)	4 (2.5%)	Ease depression and anxiety symptoms; down-regulate autonomic (sympathetic) arousal; promote neuroplasticity; modulate inflammation and immune activation
Escitalopram SSRI	1 (3.8%)	11 (7.00%)	Ease depression and anxiety symptoms; down-regulate autonomic (sympathetic) arousal; promote neuroplasticity; modulate inflammation and immune activation
Duloxetine (SNRI)	1 (0.6%)	1 (0.6%)	Ease depression and anxiety symptoms; modulate inflammation and immune activation
Venlafaxine (SNRI)	1 (0.6%)	1 (0.6%)	Ease depression and anxiety symptoms; modulate inflammation and immune activation
Mirtazapine (NaSSA)	1 (0.6%)	6 (3.8%)	Ease depression and anxiety symptoms
Moclobemide (reversible MAOI)	1 (0.6%)	1 (0.6%)	Ease depression and anxiety symptoms
Atypical antipsychotics, mood stabilizers, typical antipsychotics, and anticholinergics for antipsychotic-mediated dystonic symptoms			
Any antipsychotic	35 (28.5%)	73 (46.2%)	
Quetiapine	26 (16.5%)	60 38.0%)	Small, night-time dose to improve sleep if the other options (melatonin and clonidine) have failed to re-set the circadian clock. Time limited. PRN dose to down-regulate arousal (e.g., on return to school)
Olanzapine	4 (2.5%)	12 (7.6%)	PRN as part of FS safety plan (FS > 60 minutes) (n = 6) PRN as part of functional movement safety plan (n=1) PRN to down-regulate arousal (n = 3) PRN for sleep (n = 1) Eating disorder management (n = 1)
Risperidone	6 (3.8%)	3 (1.9%)	PRN to down-regulate arousal (n =1) Tourettes Disorder management (n = 1) Sleep management (n=1)
Aripiprazole	1 (0.6%)	2 (1.3%)	Irritability and aggression in child with autism (n = 1) Irritability and aggression in child with autism with comorbid bipolar disorder (n = 1)
Pericyazine	1 (0.6%)	1 (0.6%)	Management of anxiety (prescribed by private psychiatrist)
Lithium	0	1 (0.6%)	Bipolar Disorder
Benzatropine	0	1 (0.6%)	Once month trial for functional jaw dystonia effecting eating trialled by dental team.
Benzodiazepines (not for the treatment of epilepsy)			
Diazepam	3 (1.9%)	0	Ceased because of addictive properties
Pain medications			
Any pain medication (excluding paracetamol)	18 (11.4%)	20 (12.7%)	
Paracetamol	Missing data	106 (56.5%)	PRN Panadol available for pain flareups
Ibuprofen	0	5 (3.2%)	PRN for pain flareups
Naproxen	1 (0.6)	2 (1.3%)	PRN for pain flareups
Indomethacin	1 (0.6)	0	–
Gabapentin	6 (3.8)	7 (4.4%)	Pain management (pain team)
Pregabalin	1 (0.6)	5 (3.2%)	Pain management (pain team)
Tramadol	0	1 (0.6%)	Pain management (pain team)
Oxycodone	2 (1.3)	1 (0.6%)	Time-limited script to be used once a day prior to physiotherapy in a child with painful dystonia (pain team)
Pizotifen	2 (1.3)	2 (1.3%)	Migraine prevention (neurology)
Amitriptyline (tricyclic, used for pain management)	4 (2.5%)	0	Ceased to start an alternative antidepressant (53).
Medications for functional gut symptoms (nausea and constipation)			
Any gut medication	18 (11.4%)	28 (17.7%)	
Ondansetron	2 (1.3)	2 (1.3)	Management of nausea
Omeprazole	13 (8.2)	11 (7.0%)	Management of nausea Proton pump inhibitor
Aperients	5 (3.2%)	20 (12.7%)	Management of constipation, most typically with stool softeners such as Macrogol 3350 (Movicol) and lactulose
Hyoscine-N- butylbromide (Buscopan)	0	1 (0.6%)	Stomach cramps/pain recommended by gastroenterology team
Stimulants			
Ritalin	2 (1.3%)	2 (1.3%)	ADHD
Dexamfetamine	4 (2.5)	4 (2.5)	ADHD (n=3) Narcolepsy (n=1)
Anti-epileptics			
Any medication for managing epilepsy	13 (8.2%)	11 (7.0%)	Epilepsy management by neurology
Na valproate	9 (5.7%)	7 (4.4%)	Epilepsy management
Levetiracetam (Keppra)	2 (1.3)	1 (0.6%)	Epilepsy management
Carbamazepine	1 (0.6%)	1 (0.6%)	Epilepsy management
Oxcarbazepine	1 (0.6%)	1 (0.6%)	Epilepsy management
Lamotrigine	2 (1.3)	2 (1.3%)	Epilepsy management
Clobazam	1 (0.6%)	0	Ceased as per neurology change-of-medication plan
Cannabidiol (CBD)	1 (0.6%)	1 (0.6%)	Epilepsy management
Ethosuximide	2 (1.3%)	2 (1.3%)	Epilepsy management
Brivaracetam	1 (0.6%)	0	Epilepsy management
Lacosamide	1 (0.6%)	1 (0.6%)	Epilepsy management
Amantadine	1 (0.6%)	1 (0.6%)	Part of neurology treatment plan trial for treatment resistant epilepsy
Asthma medications			
Fluticasone propionate (Flixotide puffer)	6 (3.8)	6 (3.8)	Asthma management
Montelukast	1 (0.6%)	0	Ceased due to potential contribution to patient’s suicidal ideation
Food Supplements			
Iron	6 (3.8)	17 (10.8%)	Management of low iron levels after blood screening
Vit D	9 (5.7)	18 (11.4%)	Management of low Vit D levels after blood screening
Vit B12	8 (5.1)	10 (6.3%)	Management of low B12 levels after blood screening
Salt tablets	2 (1.3)	5 (3.2%)	Management of POTS after a positive diagnosis following a standing test (22).
Miscellaneous			
Baclofen	1 (0.6%)	1 (0.6%)	Prescribed by referring neurologist for intermittent dystonia in hips and feet, reported to be helpful by the child and was left unchanged
Furosemide	1 (0.6)	1 (0.6)	Idiopathic increased intracranial pressure, prescribed by neurology
Isotretinoin	3 (1.9)	3 (1.9)	Treatment of acne
Metformin	2 (1.3%)	2 (1.3%)	Anti diabetic agent for pre-diabetes due to weight centiles of 94.5 and > 99.0.
Oral contraceptive pill (OCP)	1 (0.6%)	5 (3.2%)	Menstrual management for menorrhagia, irregular periods. Patients were not yet sexually active.
Thyroxine	2 (1.3%)	2 (1.3%)	Treatment of hypothyroidism
Tranexamic acid	0	3 (1.9)	Treatment of menorrhagia

HCN, hyperpolarization-activated cyclic nucleotide-gated) channel blocker.

NaSSA, noradrenaline and specific serotonergic agent.

POTS, postural orthostatic tachycardia syndrome.

SNRI, serotonin noradrenaline (norepinephrine) reuptake inhibitor.

### 3.4 Medication intervention during admission

During the admission, 130 (82.3%) children had a pharmacotherapy intervention: adjusting doses, or discontinuing or initiating a medication. Seventy-four (46.8%) had one intervention; 41 (25.9%) had two; 12 (7.6%) had three; and 3 (1.9%) had four.

### 3.5 Medication use on discharge from the Mind-Body Program

On discharge from the Mind-Body Program, the majority of children (n = 140; 88.6%; mean = 3.01; median = 3) were discharged on some sort of prescribed medication (see **Table 4**). The most common medication groups were as follows: antidepressants for anxiety or depression (n = 111; 70.3%); atypical antipsychotic medication for extreme anxiety or arousal (including difficulties falling asleep and management of functional seizures that lasted more than an hour) (n = 73; 46.2%); melatonin hormone supplement for sleep (n = 64; 40.5%); α agonists, β blockers, and HCN channel blockers for down-regulating arousal, helping with sleep initiation, or managing trauma-related nightmares or extreme HR increases in POTS (n = 58; 36.7%); iron/vitamin supplement (30; 19.0%); medications for managing functional gut symptoms (n = 28; 17.7%); and medications for managing comorbid pain (n = 20; 12.7%). While all children with comorbid pain had the option of PRN paracetamol to manage exacerbations of pain, most did not avail themselves of this, as they reported that they found it unhelpful.

In the subgroup of children with ASD (n = 19) all were medicated, and medication use in these children was higher than in those without ASD (t (156) = 3.116; p = .002). A key issue in this subgroup was the management of high arousal in the context of decreased capacity to engage in arousal-decreasing regulation strategies without medication.

In the broader subgroup of children with neurodevelopmental disability—ASD or intellectual disability (delayed or borderline IQ)—30/31 children were medicated, and medication use in these children was higher (greater number of medications) than in those without neurodevelopmental disability (t(156) = 4.224; p <.001).

Over time (from cohort 1 to cohort 2), there was a shift in the mind-body team’s use of antidepressants. In cohort 1, fluoxetine was the most commonly used selective serotonin reuptake inhibitor (SSRI), and in cohort 2, fluvoxamine was the most commonly used SSRI (χ2 = 5.11; p = 0.024) (see **Table 5**). The percentage use of other antidepressants across the groups remained unchanged.

**Table 5:** Shift in SSRI use across time.

Antidepressant	Cohort 1 (2006 – 2018) 41/64 (64.1%) were discharged on an antidepressant Number of children	Cohort 2 (2018-2024) 70/94 (74.5%) were discharged on an antidepressant Number of children
Fluoxetine	25	26
Fluvoxamine	9	27
Other	7	17

For the management of arousal, clonidine, an α-2 agonist, was the most commonly used medication (see **Table 4**). Clonidine has a short half-life in children. Peak plasma concentration occurs within 1–3 hours (34). Hence, clonidine is clinically affective for approximately 4–6 hours and needs to be taken multiple times a day. Guanfacine, an α-2a agonist, was the next most commonly used medication for managing arousal. Guanfacine has a long half-life and is taken once daily. In Australia, it is approved for the treatment of attention-deficit/hyperactivity disorder (ADHD) and, if used for other indications, is very expensive because the cost is not subsidized. Eight patients were admitted on guanfacine and were also discharged on it. Ten more were commenced on guanfacine during admission—typically after they had had a trial of clonidine. Three of these had comorbid ADHD.

## 4 Discussion

The current study reports on the adjunct use of medications in treating a cohort of children with FND admitted to the Mind-Body Program. The program is run in a tertiary care hospital that services the state of New South Wales. Children who are offered a place in the program occupy the more severe end of the illness spectrum, as reflected in the following: marked physical impairment (mean GAF score of 35.39); lost days at school (89.87%); high rates of comorbid psychiatric disorders (84.8%), functional disorders (33.6%), and medical conditions (31.0%); and high levels of distress (total DASS score) and reported ACEs (total ACE score). On admission the Mind-Body Program—a rehabilitation program for FND—60.13% of children were taking one or more medications prescribed by their family doctor or paediatrician. The most common indications were a mental health disorder or management of somatic symptoms that were part of the child’s functional presentation (e.g. sleep problems, high levels of arousal, functional gut symptoms, POTS, or comorbid pain). On discharge, 88.6% of children were taking one or more medications. The most common indications were the same as those on admission, as well as supplementation for low iron, vitamin D, and vitamin B12, found on routine blood screen (part of the admission process).

In the sections that follow, we discuss how medication is prescribed in the context of a therapeutic ritual. The therapeutic ritual helps to amplify the therapeutic response and to maximally harness the associated placebo response. We also discuss the key pharmacotherapy interventions implemented during admission to Mind-Body Program and their rationale. Throughout the discussion we use illustrative vignettes to capture the breadth, depth, and range of the clinical situations encountered and corresponding treatment approaches.

### 4.1 The use of the treatment ritual

Treatment within the Mind-body Program is a structured intervention—a treatment ritual—that the clinical team use to steer the child in the direction of health and well-being (35). The mind-body team “use the psychosocial context of the treatment program to communicate that effective care is available. [The team] create a beneficial treatment ritual—ranging from simple rituals of prescribing medication (when indicated) to complex rituals of multimodal treatment. The name, structure, and psychosocial context of the treatment program convey an implicit, but powerful message to the child and the family: “You are now part of a special program that will help you get better.” (p. 529) (35)

In this context, the ritual of prescribing medication includes positive suggestions about the utility of medication—that the medication, combined with all the other components of the treatment program—will help the child get well. Positive expectations are part of the placebo effect, which contributes to the process of healing and recovery. When treating children with FND, it is important to use positive suggestions to activate the placebo response alongside the actual physiological effects of the medication and other treatment. The positive expectations that are activated as part of the placebo response engage brain regions involved in reward processing and analgesia (36). By contrast, the nocebo response—negative expectations of the treatment—decreases activation in these regions (36). The vignette below gives an example of the nocebo response in a school-age boy with FND involving cognitive regression, loss of memory (did not know who his parents were), and a functional voice disorder (a high-pitched voice, termed *puberphonia*).

During his admission to the Mind-Body Program, Rodrigo was trialled on 6.25 mg quetiapine to see whether this medication might help his anxiety about attending the hospital school. Two minutes after swallowing the quetiapine—before the medication could be metabolised—Rodrigo reported that the medication was making him worse and that he did not want to take it.

Given the above, prescribing medication to children with FND, needs to be done as part of a therapeutic ritual. The following elements of the ritual are important:

– Creating positive expectations that the medication will be helpful, especially when combined with other therapeutic interventions.
– Explaining how the medications will help. For example, explain that medications that normalise sleep will help with healing because sleep allows communication between brain cells to be reset (restoration of synaptic strength and cellular homeostasis) (37, 38).
– Using ritualistic of dosing schedules. Start with very low doses and, in a ritualistic fashion, slowly increase them if the medication is well tolerated.
– Carefully discuss information about potential side effects and how to monitor for them, to avoid making suggestions that side effects will occur. For example, emphasize that side effects are low when dosing is started with “tiny” doses and increased slowly. Or, provide information about potential side effects directly to the parents, in a separate conversation from the child. Questions to the child about how the medication is going should be general, without prompting attention to, or suggesting the occurrence of, particular side effects.

### 4.2 Treatment of comorbid anxiety and depression

The most common pharmacotherapy intervention during the Mind-Body Program was to initiate medication for managing anxiety or depression. Previous outcome studies evaluating the Mind-Body Program have shown that functional outcomes are less favourable for children whose comorbid mental health disorders do not resolve (13). These data highlight the importance of treating comorbid mental health disorders as effectively as possible with combinations of psychotherapy and pharmacotherapy (39, 40).

In the current cohort, using an antidepressant as an adjunct to treat the child’s anxiety or depression typically involved one of the following interventions: maintaining the child on an SSRI that was already prescribed and well tolerated; washing out an activating SSRI (e.g., fluoxetine) and switching to a less-activating SSRI (e.g., fluvoxamine); or commencing, with slow up-titration, an SSRI or other antidepressant when previous antidepressants had been ineffective or had not been tolerated due to side effects.

To avoid somatic side effects—e.g., nausea or headache—all antidepressants were begun at very low doses (e.g., 2.5 mg fluoxetine or 12.5 mg fluvoxamine). If the small dose was well tolerated, it was up-titrated every 3–5 days until the therapeutic dose was reached (lowest end of the therapeutic dose). At this slow rate, up-titration typically continued into the first 2–3 weeks after discharge from the two-week Mind-Body Program.

Psychoeducation about the medications was prioritised. Children and families were informed that antidepressants took time to work and that a trial of 3–6 months was needed to gauge the medication’s utility. It was suggested—both to the family and in the discharge report to the child’s clinicians in the community—that if the antidepressant was deemed to be helping the child’s anxiety or depression on review (e.g., at three months), the child should stay on the medication for 12–24 months. During that same period the child was expected to engage in ongoing psychotherapy as a means of building up their skills and capacity for maintaining resilience. But if the medication was not deemed to be helpful during that period, the child and family, along with their clinicians, should reassess their options.

Children and families were always informed that antidepressant medications worked best when combined with psychotherapy and regular pleasurable exercise. Both these interventions facilitate neuroplasticity effects—promoted by the SSRIs—in the direction of enhanced physiological regulation, improved physical and emotional resilience, and improved cognitive function (41). The overarching idea is that the brain forms and strengthens pathways through repetition (42). Known as *Hebb’s rule*, this idea is often paraphrased via the catchy lyric “neurones that fire together, wire together” (42–44).

Another element of psychoeducation pertained to the future. Families always asked about the future possibility of relapse, both of FND and of the comorbid anxiety and depression. The waxing and waning nature of anxiety was explained, as was the need to keep the child’s skill base updated with top-up blocks of treatment (e.g., with a psychologist). It was also explained that some children might need to reconsider taking medication in the future during times of high stress or when it was necessary to manage difficult life events—especially if the anxiety or depression reared its head yet again.

Over time—cohort 1 (2006–18) versus cohort 2 (2018–24) —the choice of our first-line antidepressant changed from fluoxetine (45) to fluvoxamine (see **Table 5**). This change occurred because we had noticed that fluoxetine—despite a small starting dose and slow up-titration—was, for some patients, associated with an initial period of activation, and this activation contributed adversely to their already overactivated stress system (3)(see **Figure 2**). We noticed, for example, a number of patients where fluoxetine initiation or an increase of fluoxetine dose—alongside other stressors—appeared to have contributed to the onset or worsening of functional seizures. In these patients, withdrawing fluoxetine (or decreasing a dose that had been increased) was part of our treatment intervention. In this context, when initiating SSRIs in children with FND, we began to use fluvoxamine—which is much less activating—as our first-line SSRI. Fluvoxamine also has the advantage of enabling the prescriber to use a very small initiating dose (e.g., 12.5 mg) and to easily titrate up in equally small doses (12.5 mg), thereby avoiding adverse side effects in the form of additional somatic symptoms. The issue of somatic side effects is of particular importance in patients with FND who, at baseline, present with comorbid nonspecific functional symptoms (77.8% of the total cohort) or comorbid functional syndromes (33.6% of the total cohort).

If the child’s anxiety symptoms were particularly disabling, SSRI initiation was preceded by small doses of quetiapine (6.25–12.5 mg) to help reduce the child’s anxiety symptoms until a therapeutic response from the SSRI had begun. Initiating only one medication at a time also avoided any possible confusion about potential side effects. Quetiapine was used in small doses across the day (e.g., 6.25 mg at 8 a.m., 12 noon, and 4 p.m.) to down-regulate arousal so that the child could get on with the treatment program. Quetiapine (e.g., 6.25 mg, with the option of titrating up to 25 mg) was also used at night for sleep initiation if melatonin or melatonin plus clonidine had not helped for that purpose. The child and family were always informed that the use of quetiapine was temporary, that the team was using it because of its immediate calming effects, but that long-term use needed to be avoided, if possible, because of the medication’s metabolic side effects. Longer-term use was nevertheless required to treat severe mood disorders or psychosis.

The following vignette of Nancy (pseudonym) provides an example of how medications for depression were used to scaffold the child to enable her to engage in the treatment intervention.

Following the COVID-19 vaccination, 13-year-old Nancy had developed autonomic-system dysregulation that manifest as POTS and functional gut symptoms (pain, nausea, and constipation), coupled with tension headaches and fatigue. She subsequently suffered from a severe urinary tract infection with fevers, vomiting, and weight loss. At this time she was noted to be iron deficient secondary to heavy periods. While still recovering from the infection, Nancy developed visual symptoms and tremors in her legs. The tremors progressed to leg weakness and occasional locking of one knee. After a thorough medical workup, she was diagnosed with FND (with motor and sensory [visual] symptoms).
The local paediatric team ran a rehabilitation admission at the local rural hospital. The admission did not go well. The team and the family reached an impasse. The local team reached out for help. On discussion of the problems, the rural team complained that Nancy was uncooperative and that the family had complained that the team had been punitive, setting expectations that Nancy was unable to manage. After the usual mind-body assessment at our tertiary-care hospital, Nancy was offered a two-week admission into the Mind-Body Program.
In the first week of the admission, the mind-body team noticed that Nancy suffered from a severe major depression—flat affect, low mood, anhedonia, sleep disturbance, difficulties concentrating, amotivation, and inner sense of emptiness—that made it difficult for her to engage in any of the program. The depression had not been treated. Fluoxetine had been initiated just weeks before and had yet to reach a therapeutic dose. Moreover, a routine blood screen showed low iron and vitamin D levels—both known to contribute to symptoms of fatigue. When Nancy developed gastroenteritis during the second week of the admission—and had to be discharged home to recover—we negotiated with the family to time Nancy’s re-admission some weeks hence, once Nancy’s depression had lifted somewhat. We up-titrated the dose of fluoxetine and added quetiapine at night (up-titrated to 50 mg) to manage Nancy’s disrupted sleep and negative ruminations that occurred at bedtime. We also prescribed iron and vitamin D supplements. Since Nancy had struggled to engage with her local psychologist, we supported a block of equine therapy (it was available in her rural setting). We then reviewed Nancy (and family) regularly via online sessions.
Six weeks later, when Nancy began to joke with the team as to which of her father’s horror-figurine collection she should bring with her to her second mind-body admission—to scare the team—it was clear that she was ready for another try. On return to the Mind-Body Program, Nancy, who still suffered from leg weakness and an abnormal gait, engaged in all aspects of the program. After two weeks of hard work, she was discharged mobilizing without aids. After a month of outpatient physiotherapy, she had returned back to school full time and was participating in her weekly dancing classes. The quetiapine was withdrawn some months later, and it was recommended that Nancy remain on the fluoxetine for a period of 12–24 months while she engaged in therapy to strengthen her emotional and physical capacities and to improve her well-being.

The vignette of Elizabetta describes the treatment of a depression that is much more treatment resistant and that adversely affected the recovery process, which lasted for many months.

Elizabetta was a 12-year-old girl who presented to the emergency department with weakness in all limbs after a fall and a two-year history of bullying and social rejection at school where other students mocked her dedication to learning. Medical investigations revealed no injuries. Three days later, Elizabetta’s weakness progressed to paralysis of her lower limbs; fluctuating paralysis of her right arm; and pain in her neck that caused her body to tremor. A comprehensive neurology workup (including EEG) was completed, and a diagnosis of FND was given. During assessment for the Mind-Body Program, Elizabetta reported a prolonged history of low mood (0/10) most days; difficulties with concentration and motivation in the classroom; sleep-onset difficulties and recurrent waking throughout the night; persistent fatigue; and reduced appetite. Observationally, throughout the assessment Elizabetta presented with blunted affect. She reported that her mood had begun dropping several years earlier when several girls in her class started bullying her (including physical bullying in the form of slapping, pinching, pushing, and so on).
Elizabetta was commenced on 25 mg of fluvoxamine, up-titrating to 150 mg over a three-month period. Because Elizabetta did not recover in her first two-week mind-body admission, she was offered a series 1- or 2-week inpatient blocks, with weekly outpatient psychotherapy and physiotherapy sessions, along with online access to the hospital school when she was not admitted to hospital. She made little progress and was wheelchair dependent during this period.
As Elizabetta’s depressive symptoms started to improve, she experienced an increase in social anxiety—including panic attacks—which would at times morph into functional seizures. Each time the team discussed return to home school or an outing in a public space, Elizabetta experienced panic attacks due to worries of people staring at her in the wheelchair. Elizabetta’s fluvoxamine was up-titrated to 200 mg a day, and quetiapine 25 mg three times a day was introduced to help down-regulate her stress system.
It took over 12 months of psychotherapy and pharmacotherapy to address Elizabetta’s mood and anxiety symptoms to a level where she could actively engage in mind-body rehabilitation and make functional gains. Eighteen months after presentation, all of Elizabetta’s FND, mood, and anxiety symptoms had resolved. She was able to mobilise independently, and she had returned to normal school. Her quetiapine was withdrawn, and it was recommended that she stay on fluoxetine for another 12–24 months while engaging in psychotherapy to build up her repertoire of skills to manage academic stress and the stress of negative peer relationships. A subsequent referral for formal assessment confirmed a diagnosis of ASD, which had been suspected by the mind-body team.

### 4.3 Regulation of sleep and the circadian cycle

Disruption of the sleep cycle is common in children with FND (11). A subset of children in the current cohort (n = 32) participated in a study looking at their cortisol awakening response (11). Compared to healthy controls, the children with FND showed an attenuated CAR or, in some children, an obliterated/reversed CAR. Unsurprisingly, the second most common pharmacotherapy intervention during the mind-body admission was the use melatonin to stabilise the circadian cycle. Melatonin was always used alongside behavioural interventions that included a timetable with a formal bedtime, exposure to sunlight in the morning (blinds up), and implementation of sleep hygiene measures.

Amalia was a 12-year-old girl whose ambition was to be a gymnast. Eight months ago, Amalia had needed to wear an orthopaedic neck collar after landing badly in a fall. One month ago, she again twisted her neck in a fall. She continued to experience headache and fatigue despite normal medical investigations. She then developed leg weakness and an unsteady gait, which were diagnosed as FND. She was admitted to the Mind-Body Program (daily physiotherapy, psychotherapy, hospital school, and weekly family sessions). She started on melatonin to help manage her disturbed sleep, and she learnt, and began to implement, specific strategies to manage her pain. During the admission, Amalia’s sleep improved, her capacity to manage her pain was bolstered, and her walking difficulties resolved. Amalia then returned to school and continued working with a psychologist to improve and maintain her mind-body regulation strategies. No other contributing factors were identified in Amalia’s history.

For children with a reversed circadian clock, a shift of the circadian rhythm to a more normal one was usually attained prior to admission by going to bed two hours later each day or by staying up for a night and a day to reset the circadian clock.

Fifteen-year-old David was very distressed by his functional somatic symptoms—migrating pain worse in the back, and right leg weakness and loss of sensation—and he was highly motivated to be accepted into the inpatient Mind-Body Program. In establishing a treatment contract with David and his mother, the team explained that the program was hard work and that before beginning the program, David needed to engage in some preliminary interventions. First, he needed to return his sleep cycle to a normal rhythm. He could do this over a two-week period by going to bed two hours later each day, until he got to his previous healthy bedtime of 10 p.m. Once he reached that point, the team wanted him to take melatonin, a natural substance that the brain secretes to help with sleep, so that his sleep cycle stayed regulated. They also wanted him to eat breakfast in a sunny spot in the house, making sure he got a good dose of morning light. Second, David needed to eat three healthy meals a day—including sufficient vegetables, fruit, and yogurt—to make sure that he had energy for the program and that he was looking after his microbiome, the bacterial community in his gut, which plays a key role in body regulation. Third, David needed to restart the antidepressant (an SSRI) that had helped him previously, because the team would be unable to work with him if his mood remained low. In addition, since antidepressants improve the brain’s plasticity, they would help his body reset pain set-points and so on. Fourth, he needed to go outside the house every day. At first, he could mobilize to the front gate, but he had to increase the distance by a minimum of two metres a day. He also needed to re-engage with his physiotherapist. These pre-program interventions would prepare him for the rigours of the Mind-Body Program. David implemented all the above interventions over a six-week period. Subsequently, after his two-week inpatient admission, he was walking with a normal gait, and his pain had decreased significantly. He maintained his improvements over the summer holidays, after which he returned to school full time and initiated ongoing therapy (reproduced from Kozlowska et al., 2020 (6)).

### 4.4 Using medications to down-regulate arousal, manage POTS and trauma-related nightmares

Children with FND—and especially the subgroup with functional seizures—have high levels of brain and body arousal reflected in autonomic system activation (21, 46) and EEG measures of cortical activation (3). In this context it is not surprising that, in the present study, the third most common pharmacotherapy intervention during the mind-body admission was the introduction of arousal-decreasing medications to mitigate the high-arousal conditions under which FND symptoms occur, and to manage comorbid POTS and trauma-related nightmares.

**Figure 4:**
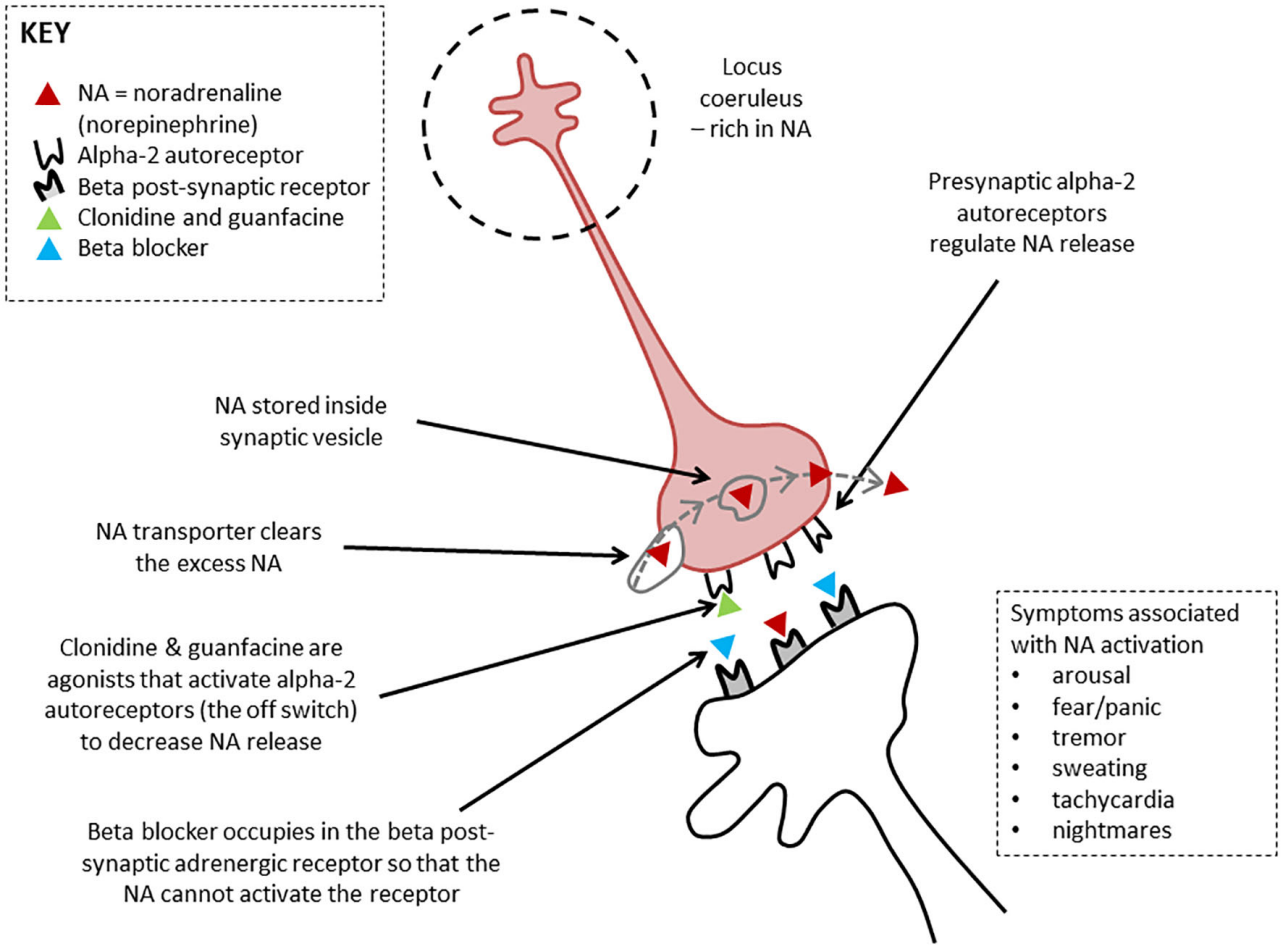
Therapeutic actions of clonidine, guanfacine, and beta blockers. Noradrenergic neurons mediating brain arousal from the locus coeruleus. The locus coeruleus is the main source of noradrenaline synthesis in the brain. It reaches upward, forward, and downward, sending projections throughout the brain. Clonidine and guanfacine are agonists that activate alpha-2 autoreceptors (the off switch) to decrease noradrenalin release. Beta blockers occupy the beta post-synaptic adrenergic receptor, effectively blocking it so that the noradrenalin cannot activate the receptor. Figure originally developed for Savage and colleagues, 2022 (33). ^©^ Kasia Kozlowska 2022.

Clonidine, a nonselective α-2 agonist, was the medication most frequently used at doses of 25–350 μg a day. Clonidine can be used in small doses across the day (e.g., 25 μg at 8 a.m., noon, and 4 p.m.) to down-regulate arousal. Through its α-2 agonist activity at autoreceptors (see **Figure 4**), clonidine functions as a break for the locus coeruleus, a nucleus in the brainstem. The mass of noradrenaline-containing projections emanating from the locus coeruleus and reaching throughout the brain modulate noradrenalin levels across brain networks (47, 48). Clonidine puts a break on this system—down-regulates noradrenalin (catecholamine) production—thereby decreasing brain network arousal.

**Figure 5:**
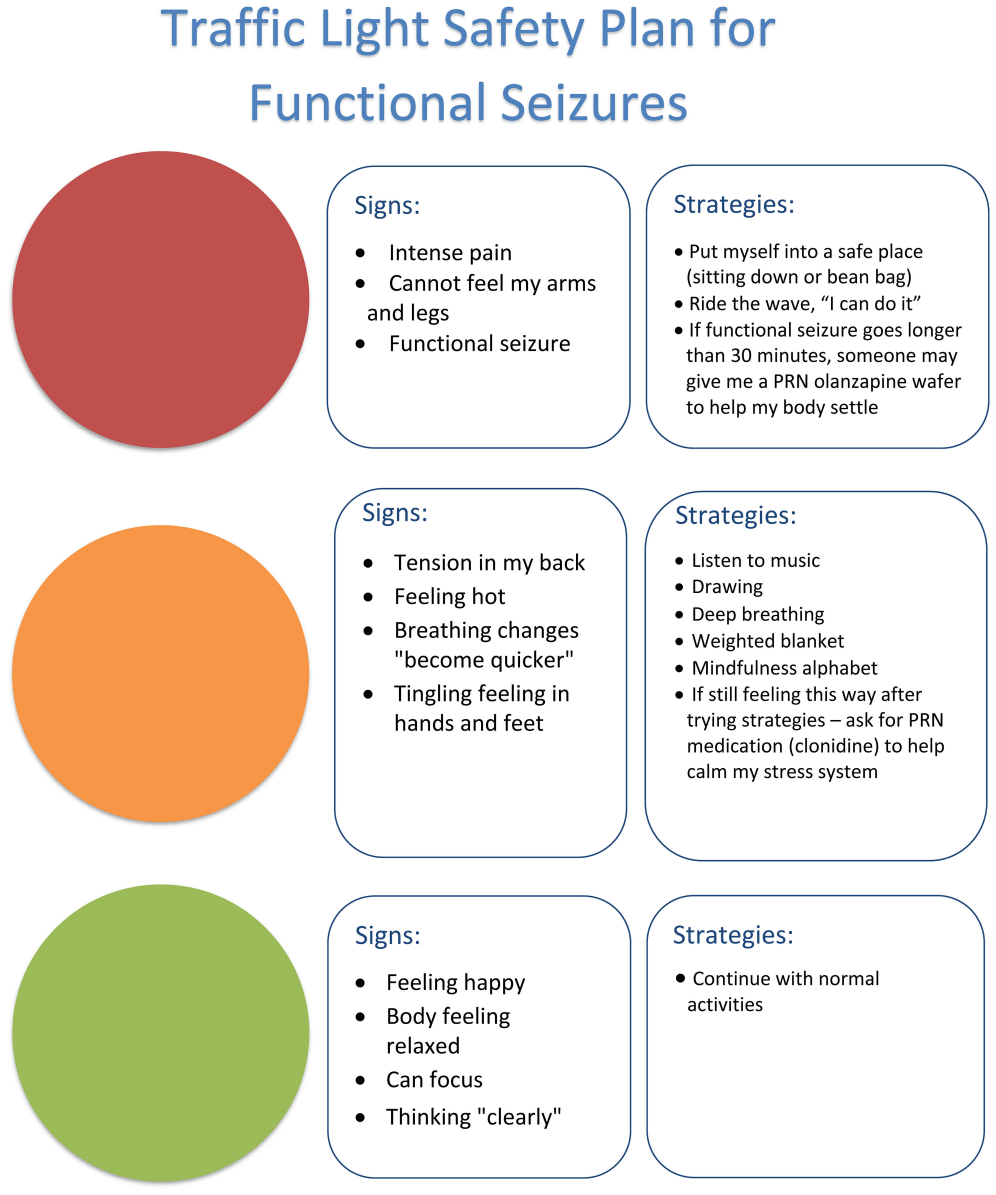
Traffic light safety plan ^©^ Danae Laskowski & Kasia Kozlowska 2019.

In the Mind-body Program, the use of small regular doses of clonidine is always coupled with implementation of regulation strategies that the child learns and practices on a daily basis in order to build the skill base that will, in time, enable them to down-regulate arousal without needing to use any medication. In combination, the clonidine and the regulation skills acquired by the child decrease the probability that functional neurological symptoms—functional seizures, functional tic attacks, and so on—will be activated, and they provide the child with skills to calm down when such symptoms do arise. When FND symptoms prove to be especially difficult to contain, clonidine can also be used PRN (e.g., 25–50 μg) to support the child, in combination with their learned mind-body strategies, to manage escalations of arousal. An example is the safety plan for functional seizures, where the child learns to read their state of arousal and to implement strategies to help the brain and body to calm down, so as to prevent the onset of a functional seizure (see **Figure 5**). In cases where the child is struggling to down-regulate using mind-body strategies alone, clonidine PRN can be used as an adjunct.

Clonidine can also be used at night at doses of 25–150 μg to help with sleep initiation.

Clonidine tablets are soluble in water when doses smaller than 25 μg are sufficient (e.g., 10 μg four times a day) (49, 50). Dosing typically begins, however, with 25 μg, which can be titrated up when necessary to achieve a therapeutic response. Blood pressure should be monitored at larger doses. In hot climates it is important to maintain good hydration. Paradoxical effects are rare but present as a paradoxical increase in agitation.

In the present study, a subset of 10 children trialled on clonidine were crossed over to a long-acting α-2a agonist guanfacine (doses of 1–4 mg a day in the morning or at night), whichever was preferred by the child). The high cost of guanfacine limited availability of this intervention. The following vignette describes the use of guanfacine to decrease arousal.

Jamie, a 14-year-old girl was diagnosed with functional tics, with a background of anxiety, depression, and friendship difficulties. During COVID, Jamie’s parents separated, resulting in increased stress in the household, in response to which Jamie spent progressively more time on social media to escape the tension in the family home. When lockdown restrictions began to ease, Jamie struggled to return to face-to-face schooling. She developed a tic-like movement (head jerking sideways) and vocalisations that emerged most mornings prior to leaving for school. Over several days, the jerking movement intensified, ultimately involving all four limbs. Jamie also started to experience violent tic attacks characterised by forceful jerking of her limbs, with one specific attack resulting in a hairline fracture of her left arm due to her arm forcefully hitting a brick wall. Jamie’s mother brought her to hospital assessment: the neurology team diagnosed FND and referred Jamie to the mind-body team for treatment. Jamie engaged well in the Mind-Body Program, but she struggled to manage her functional tics. She was therefore started on 1 mg guanfacine at night, which the team up-titrated to 2 mg after three nights in an effort to down-regulated her stress system (level of autonomic arousal). Over the next week, Jamie and the team noticed a substantial reduction in her functional tics, the majority of which Jamie was able to control using deep-breathing techniques. On the rare occasions when the use of those techniques failed to down-regulate her stress system—resulting in a tic attack—she was given an additional 1.25 mg olanzapine wafer. After discharge Jamie was linked in with an ongoing individual psychologist, and her family also engaged in family therapy to help Jamie (and the family) navigate the painful feelings associate with the parents’ separation.

The following vignette illustrates the use of clonidine to down-regulate arousal in a child with limited capacity for acquiring and independently implementing non-pharmacological regulation strategies.

Petter, a 13-year-old boy with a moderate intellectual disability, had a history of repeat presentations to the emergency department for functional seizures. Following a comprehensive neurology assessment, he was referred to the mind-body team and was admitted for a two-week mind-body admission. We learned from Petter’s class teacher that his functional seizures had started when a new student with behavioural dysregulation and loud vocalizations had joined the support classroom.
Petter was started on 25 μg clonidine three times a day (8 a.m., noon, and 3 p.m.). He tolerated this well, and the dose was increased to 50 μg three times a day. Teaching staff in the hospital school noticed that Petter would clutch his head when he started to feel overwhelmed. If the overwhelming emotion continued, Petter would have a functional seizure. A safety plan for functional seizures was developed. If the teacher (or parent) observed Petter clutch his head, he was directed to a “calm corner” with a beanbag (set up both at school and at home), where he was able to listen to music using his headphone and where he could down-regulate himself by the use of fidget toys or a weighted blanket. By the second week, Petter was attending hospital school for all sessions and experienced no functional seizures. After the admission, Petter remained on the clonidine and the “calm corner” and regulation strategy options were re-created in his home school. Following discharge, Petter had no further functional seizures.

For children in the present study who experienced severe POTS or unmanageable symptoms of panic—symptoms caused by autonomic activation and dysregulation—propranolol, a β blocker, was most commonly trialled. Propranolol is a β-adrenoceptor antagonist that blocks β-adrenergic receptors in body tissues and in the central nervous system (see **Figure 4**) (51). Adrenergic receptors are the targets of catecholamines—adrenaline and noradrenaline—which are released in response to stress-related sympathetic activation of the adrenal glands. Propranolol blocks these β-adrenergic receptors to attenuate episodes of extreme sympathetic activation that occur in the context of POTS or panic attacks. Because propranolol is lipophilic—that is, it dissolves in fat—it can cross the blood-brain barrier and act on β-adrenergic receptors in the brain, thereby decreasing arousal in brain networks (47). Propranolol is given on a morning and afternoon dosage schedule (beginning at a dose of 2.5 mg and potentially titrated up to 5 mg, 7.5 mg, or 10 mg). It is not taken at night because it can disrupt the circadian clock. Propranolol is contraindicated in young people with asthma.

Other interventions used in the study to manage POTS included increased fluid intake, compression stockings, increased intake of salt, slow-paced breathing to down-regulate autonomic arousal, and daily physiotherapy. The long-term plan was to withdraw the propranolol once the POTS symptoms had settled.

In one child with very severe POTS for whom propranolol had not been helpful, ivabradine was trialled. Ivabradine is an HCN channel blocker that decreases HR by inhibiting the cardiac pacemaker current.

The vignette of Lieta highlights the challenges of managing severe POTS.

Lieta had experienced many stressors over a period of two years. At 15 years of age, she presented to hospital with intermittent bilateral leg weakness, a persistent headache, and musculoskeletal pain that migrated to different parts of her body. Because of her symptoms, Lieta had stopped going to school, and she had been spending more and time in her bed. With time she had developed symptoms of dizziness—especially on standing up from bed—and fatigue, as well as intermittent nausea. She was referred to the Mind-body Program by the pain team, who was concerned that she was not improving with outpatient intervention. A standing test on admission was positive for POTS, with an HR increase of >40 beats per minute from lying to standing (See **Table 6**) (22). Because the POTS was very debilitating—the dizziness contributed to Lieta’s difficulties in mobilising—she was begun on a regimen of increased fluid intake, salt tablets, wearing pressure stockings during the day, implementing slow-breathing strategies that help upregulate parasympathetic function and decrease sympathetic function (dysregulated in POTS), and propranolol. The propranolol was given in the following doses: 2.5 mg in the morning, 2.5 mg at lunchtime, and 2.5 mg at 4 p.m. A night dose was avoided so as not to disrupt the circadian cycle. It was subsequently increased to 5 mg in the morning, 5 mg at lunchtime, and 2.5 mg at 4 p.m. Many months later, once the POTS had completely resolved—as had Lieta’s pain, fatigue, and FND symptoms—the propranolol was titrated down by Lieta’s family doctor. Based on the rapidity with which Lieta’s body had deconditioned, it was suggested that she continue an ongoing program of regular pleasurable exercise to ensure that she maintained her physical conditioning and continued to build on her hard-won resilience.

**Table 6:** Lieta’s blood pressure and heart rate values on the standing test for POTS.

Time period	Blood pressure (mmHg)	Heart rate (beats per minute)
Baseline (lying Down)	118/69	86
1 minute (standing)	124/80	136
2 minutes (standing)	130/86	127
3 minutes (standing)	130/86	127
4 minutes (standing)	128/82	133
5 minutes (standing)	112/79	125
6 minutes (standing)	112/88	146
7 minutes (standing)	125/85	130
8 minutes (standing)	95/67	128
9 minutes (standing)	115/90	128
10 minutes (standing)	120/88	125

Prazosin, a competitive α-1 antagonist, was used in three children for trauma-related nightmares (as part of PTSD). Prazosin targets the thalamus, leading to sedation together with simultaneous blockade of muscarinic, cholinergic, and histamine receptors in the reticular activating arousal system (48). See, for example, the vignette of Xenia.

Xenia was a 14-year-old girl presenting with FND—leg weakness, nausea, dizziness, and functional seizures presenting as blackouts—following a netball injury. Xenia, who lived with her grandparents, met diagnostic criteria PTSD, manifesting as nightmares and intrusive memories during the day, and POTS, which caused symptoms of dizziness and nausea on standing.
Xenia had experienced high levels of cumulative stress from three years of age. She, along with her siblings, had experienced the following: neglect (insufficient food coupled with a lack of appropriate adult supervision); repeated exposure to domestic violence (with fears that her siblings or mother would be irreparably injured by one of her mother’s various male partners); and repeated house moves (making it difficult to maintain a friendship group and to keep up with academic demands at school). On moving in with her grandparents three years earlier, Xenia suffered from trauma-related nightmares and hypervigilance to threat cues (symptoms of PTSD). These symptoms settled over a 12-month period, and Xenia was able to push the memories out of her mind. She was also able to make good progress at school.
Three weeks prior to Xenia’s presentation with FND, she was reading the newspaper and saw a picture of one of the men—an ex-partner of her mother—who had been arrested by the local police. The picture triggered a cascade of trauma-related memories. The memories emerged — night after night — in the form of nightmares. Three weeks later, a netball injury triggered various functional symptoms, including her functional seizures.
Xenia was admitted into the Mind-body Program. Her nightmares were treated using prazosin (starting at 1 mg at night and titrating up to 3 mg). She was also prescribed melatonin 3 mg (immediate release) and melatonin 2 mg (slow release) because—together with the prazosin—this improved her quality of sleep. Following the inpatient intervention, Xenia was referred for long-term psychotherapy—with a trauma-focused component—to process some of the trauma-related memories that continued to activate her stress system on a daily basis. (See Savage and colleagues [2022] for an earlier version of this vignette (33)).

### 4.5 Use of atypical antipsychotics to down-regulate overwhelming arousal/anxiety and as an adjunctive for depression

The fourth most common pharmacotherapy intervention in our study was the time-limited use of atypical antipsychotics. Small doses of quetiapine (0.65–12.5 mg three times a day) were sometimes used across the day to help contain high states of arousal or anxiety prior to the time that an SSRI had reached a therapeutic dose or its therapeutic effect (6–8 weeks lag time). Small doses of quetiapine were sometimes used at night in resetting the circadian clock, particularly if melatonin or clonidine had not been helpful. For examples, see the vignette of Aviva (below) and Nancy (above; section 4.2).

Aviva was a 10-year-old girl with a functional dystonia manifesting as a painful torticollis. Even when the torticollis had resolved, she continued to experience pain. Aviva was also very fearful of moving her neck in case the dystonia happened again. Because of her fear, she struggled to engage in physiotherapy, would not remove the soft collar that had been prescribed by the orthopaedic team, and had failed all efforts to return to school. Her home team had prescribed small doses of clonidine to help manage her arousal (50 μg, midday and nighttime). The clonidine helped a little, but Aviva, her family, and the treating team felt very stuck.
After the formal assessment meeting with Aviva, her parents, and the mind-body team, Aviva pronounced that she wanted to come into the Mind-body Program to give treatment another go. Because Aviva’s fear and anxiety were so intense, it was agreed that she would trial small doses of quetiapine—6.25 mg four times a day—to contain her anxiety. Because nighttime was also a difficult period for Aviva—she woke in the early morning hours of each day in pain and distress, and was often unable to get back to sleep—Aviva was provided with a “night-time rescue plan.” The rescue plan involved taking paracetamol (500 mg) for the pain and quetiapine (12.5 mg) for the distress, as a combined dose. With this pharmacotherapeutic scaffolding, Aviva was able to engage in all components of the program: physiotherapy, school, and her individual therapy, where she worked on regulation strategies that she would use to manage her pain and fear when she returned home.
During the mind-body admission, Aviva’s anxiety—including the extreme nature of her perfectionism—and her underlying struggle with low mood were noted by multiple clinicians. Because uncontained anxiety was likely to be an ongoing problem, toward the end of the admission Aviva was started on an antianxiety medication fluvoxamine (12.5 mg twice a day). The goal was to build up to a therapeutic dose of 75 mg a day over a period of a month and to then, some months later, to wean her off the quetiapine once the fluvoxamine was having a therapeutic effect. Alongside theses various interventions, Aviva’s parents had booked themselves into a course of family therapy to address some of the family-related issues that were contributing to Aviva’s stress and anxiety.

Olanzapine (wafer, 1.25–2.5 mg) or risperidone syrup or tablets (0.25–1.00 mg) were used PRN in a subset of children as part of the child’s functional seizure safety plan or tic-attack safety plan (see **Figure 5**, which depicts clonidine as the PRN medication). The children in question were typically those experiencing functional seizures of long duration (e.g., >30 minutes or so) or functional seizure/functional tic episodes that were very violent with identified risk of harm to the child or to someone else. See the following vignette of Gadin.

Gadin, a 13-year-old boy, was admitted to the Mind-body Program for treatment of functional seizures. His history included social anxiety secondary to bullying and also academic difficulties due to a specific learning disorder. Gadin had been prescribed an SSRI (fluvoxamine) for his anxiety. Within the hospital context—including hospital school—Gadin was able to read his body for functional seizure “warning signs” (hyperventilation) and to use his regulation strategies (diaphragmatic breathing) to down-regulate his stress system, thereby to averting his functional seizures.
Gadin was unable to successfully implement his new-found skills after discharge, specifically in the context of returning to his local school. On the days that he attempted to return to school, he experienced functional seizures either at home getting ready for school or in the car on the way to school. Gadin was re-admitted to the Mind-body Program. Quetiapine—25 mg three times a day—was prescribed to provide additional cover to reduce his overwhelming anxiety. Gadin and his parents also agreed to an olanzapine wafer (2.5 mg) as a “circuit breaker” to be administered during his functional seizures (see next paragraph).
In his psychology sessions Gadin engaged in imaginal exposure depicting his return to his home school. In one of these sessions, he experienced a functional seizure that continued over 45 minutes. Half an olanzapine wafer—2.5 mg—was placed inside his cheek while he was still experiencing the functional seizure. This allowed Gadin’s stress system to down-regulate, disrupting the seizure process (hence, “circuit breaker”) and to continue with his imaginal exposure sessions. Prior to attempting re-integration yet again, Gadin’s psychologist worked with the school to make sure that they implemented an individualised learning plan that took Gadin’s learning difficulties into account.
After discharge, Gadin required the circuit-breaker medication only on three occasions. With revised expectations at school (the individualised learning plan) and a graded exposure plan, he was able to return to full-time school over a period of four months. The quetiapine that had been prescribed to scaffold his efforts to manage his anxiety was weaned slowly 5–8 months later.

Quetiapine, olanzapine, or risperidone were sometimes made available to the children in our study, to be taken on their way to school when they were practicing reintegration to their local school. This use was usually reserved for children whose anxiety was so overwhelming that that efforts to reintegrate to school resulted in any of the following: running away in fear (activation of the flight response); strong avoidance that made it difficult to pry the child out of the car; disabling panic attacks; or panic-induced functional seizures. See the following vignette of Jennifer, below.

Jennifer, a 16-year-old girl, was diagnosed with FND. Her history included acute disseminated encephalomyelitis (many years before), anxiety, and learning difficulties. Jennifer suffered from episodes of intermittent leg weakness that occurred most commonly in the morning prior to a going to school and sometimes even the night before. Jennifer was engaged in cognitive-behavioural therapy. Six months prior to entering the Mind-Body Program, she had been prescribed 20 mg fluoxetine to help with her anxiety. Despite working hard with her psychologist, Jennifer continued to experience frequent bouts of leg weakness, and her school attendance had been affected. The negotiated goal in the Mind-Body Program (two weeks) was to complete one week of school in the hospital setting and for her to attend her local school during the second week of admission. Throughout the first week, Jennifer attended the hospital school with minimal difficulty and experienced no functional leg weakness. On the first day of the second week, Jennifer had difficulty mobilizing. To reduce Jennifer’s anxiety on the subsequent morning, she was prescribed a 0.25 mg dose of risperidone. This dose sufficiently reduced her anxiety to enable her to attend school without experiencing leg weakness. Jeniffer continued to utilize PRN risperidone on the morning of school throughout the remainder of the hospital admission, and also after discharge. On review two weeks later, Jennifer and her family reported she had successfully transitioned back to school full time, with only one morning of functional leg weakness. Given this success, the team provided a titration plan to reduce the PRN risperidone in the morning before school, until Jennifer was able to attend school with no PRN medication.

### 4.6 Use of supplements

The fifth most common intervention in our study was the introduction of supplements—iron, vitamin D, and vitamin B12—for children whose routine blood screen revealed these deficiencies. This intervention was coupled with education about diet and also about the need to spend time outside in the sun for vitamin D production.

Vaanya was 16-year-old girl presenting with epilepsy and a two-year history of panic attacks, episodes of functional nausea and vomiting (all reflecting autonomic activation), shaking, and ongoing symptoms of fatigue. Vaanya’ symptoms had emerged in the context of ongoing stress in the family system, including recurrent cycles of domestic violence (no longer occurring). More recently the episodes of shaking had developed into functional seizures, and Vaanya had stopped attending school. She hoped that the Mind-body Program would help her gain control of her functional seizures and enable her return to school. Routine blood screen during admission showed low levels of iron, vitamin D, vitamin B12. Since severe fatigue was a key element of Vaanya’s struggle to return to health and well-being, supplements for all three were prescribed (with subsequent monitoring by the family doctor). Other medications on discharge were sodium valproate and lamotrigine (both prescribed for epilepsy by the neurologist); moclobemide (prescribed for mood by the community psychiatrist, given that Vaanya did not tolerate SSRIs); melatonin for sleep (added during admission to stabilise her circadian clock); and PRN quetiapine (added during admission to be used as part of her functional seizure safety plan, for functional seizures >1 hour duration). Following the two-week admission, Vaanya completed a successful reintegration into her local school (including participation in school sports). When her iron, vitamin D, and vitamin B12 returned to normal, her symptom of fatigue was no longer a problem.

### 4.7 Use of medications to manage functional gut symptoms/syndromes

The sixth most common intervention was the use of medication to manage functional gut symptoms or syndromes. Aperients—most commonly, stool softeners such as macrogol and lactulose—were used to better manage functional constipation, which had not been adequately managed and which interfered with the child’s function and their capacity to engage in the Mind-body Program.

Thirteen-year-old Abigail—a talented dancer—presented to hospital with new-onset functional seizures and functional abdominal pain in the context of bullying at school, extreme commitment to high performance dance, and significant health anxiety relating to her symptoms. The medical team referred Abigail for assessment for admission into the Mind-body Program. On assessment it emerged that Abigail had a long history of chronic constipation and that she was—at that time—experiencing an increase of abdominal pain. On prompting, the medical team re-examined and X-rayed Abigail’s abdomen and found that she was severely constipated. She was started on a disimpaction regimen: a regular schedule of aperients. Once her constipation-related abdominal pain resolved, she was admitted to the Mind-Body Program for treatment of her functional seizures. Her aperients were continued during the admission and following discharge. During the mind-body admission, she was also started on an SSRI (for untreated anxiety). She continued to work on the anxiety psychotherapy) with a psychologist in the community.

**Figure 6:**
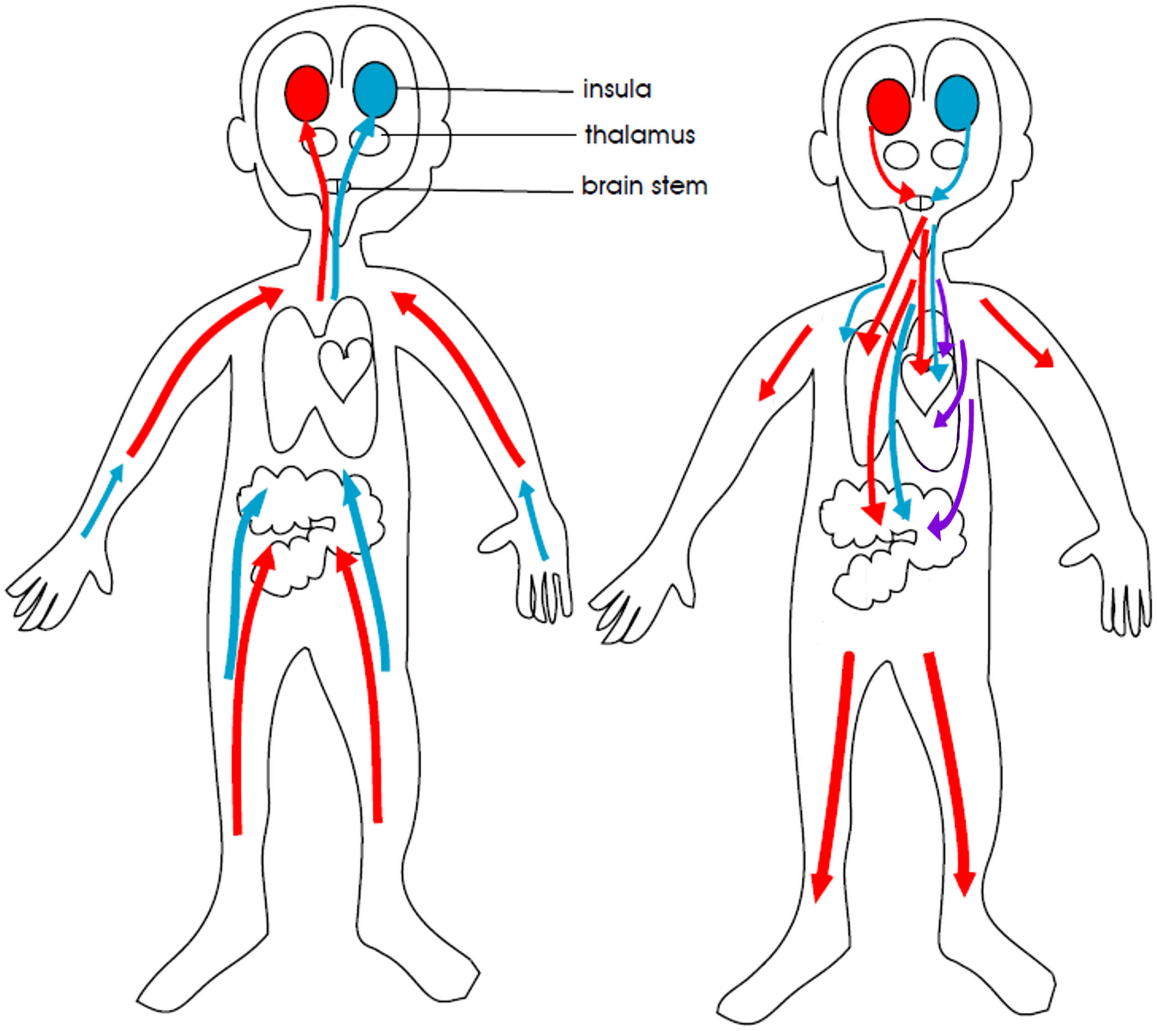
Simplified visual representation of the autonomic nervous system. The red colour represents the sympathetic nervous system, the purple colour the defensive parasympathetic system, and the blue colour the restorative parasympathetic system. The red and purple colours indicate the parts of the autonomic nervous system that activate the body and brain in response to danger and stress. The blue colour indicates the part of the autonomic nervous system that calms the body and brain. In children with FND, sympathetic (red) activity is increased. and the restorative parasympathetic (blue) activity is decreased, resulting in an overall increase in arousal. In a subgroup of children, intermittent activation of the defensive parasympathetic (purple) activity to the gut causes symptoms of nausea, vomiting, or diarrhoea (activation of defensive gut programs) or much more rarely, threat-related fainting (activation of defensive vagal fibres to the heart). ^©^ Kasia Kozlowska 2013.

Medications for gut-related functional symptoms—ondansetron (anti-nausea) and omeprazole (anti-reflux)—were used to manage debilitating nausea and reflux when interfered with the child’s capacity to engage in the Mind-body Program (see **Table 4**). In terms of psychoeducation, the family is typically told that nausea—akin to pain—is one of the body’s alarm signals that signals that something was wrong. Nausea typically settles only when the child had returned to normal function. **Figure 6** depicting the autonomic nervous system can be used to discuss the role of the defensive vagus—the purple arrow in the picture—in switching on defensive programs of nausea, vomiting, or diarrhoea in the gut (see **Figure 6**).

Following a serious influenza infection, Teodora—a 15-year-old adolescent girl—presented with astasia-abasia (loss of coordination in the legs), POTS, and exacerbation of her long-standing irritable bowel syndrome. On admission, Teodora reported that mornings were a particular struggle, “I wake up and I just feel like throwing up. It really makes it hard to get to school. And then at school I just feel nauseous all the time”. A trial of ondansetron (4 mg twice daily) helped contain Teodora’s nausea, and she was better able to engage in the Mind-Body Program, including regular attendance at the hospital school.

### 4.8 The management of pain

In the current cohort of children with FND, two-thirds experienced complex pain. At the initial assessment meeting, the psychoeducation component included the following information about the pain.

  – Pain is an alarm signal that something is wrong. In children with FND the activation of the alarm system is common.
  – The pain alarm is driven by activation of the stress system (52), which is also responsible for driving the child’s FND symptoms. In the Mind-Body Program, we help the child down-regulate the stress system via regular exercise (physiotherapy), regulation strategies (psychotherapy), and adjunct medication (e.g., clonidine).
  – Complex pain does not typically respond well to pain medications. The child is welcome to use simple analgesics if they are helpful—especially prior to physiotherapy—but otherwise we would not be using pain medications.
  – Typically, pain is one of the last symptoms to resolve. It tends to resolve when the child has regained full function. So, we focus on regaining function. Only then will the body turn off the pain alarm signal.

This de-emphasis on pain is reflected our use of pain medications (see **Table 4**). Despite the high rate of comorbid complex pain (66.5%) in our cohort, the use of prescribed pain medications (opioid-based medications, gabapentinoids (gabapentin and pregabalin), and non-steroidal anti-inflammatory medications, was low (12.7%). As a general rule, opioids and gabapentinoids were prescribed in collaboration with the pain team for a time-limited period—when the child and mind-body team were working on mobilization.

Other interventions pertaining to pain were as follows. Withdrawal of benzodiazepines (diazepam)—because of their dependence properties—that had been prescribed for pain related to dystonia, muscle spasms, or muscle tension (n = 3). Withdrawal of amitriptyline prescribed for pain when it was necessary to prescribe an SSRI to treat a child’s comorbid depression (n = 4). Amitriptyline does not address depression in children (53), and amitriptyline and SSRIs should not be combined because of the risk of serotonin syndrome.

The vignette of Pearl provides an example of an intervention where a time-limited use of oxycodone allowed the child to make progress in her physiotherapy sessions after a long period of failed mobilisation.

Pearl was 12-year-old girl who had developed complex regional pain syndrome following an injury to her left knee during a long-distance marathon event. Despite intense outpatient physiotherapy—and trials of amitriptyline (discontinued) and gabapentin (ongoing) by the pain team—Pearl resisted putting weight through her leg and became wheelchair dependent. Some months later she developed tremor episodes (in the foot), which resulted in long episodes of distress characterized by screaming, moaning, and weeping. On assessment for the Mind-Body Program, Pearl was told that the only way to stop the tremor and to decrease the pain was to regain normal function. The team made the following deal with Pearl. The team would promise to give her an oxycodone dose before her daily physiotherapy session during the two-week program—in order to support her in the task of mobilising her leg—if Pearl promised to work with the physiotherapist to put weight through her foot. The deal yielded a positive outcome. Pearl worked hard in her physiotherapy sessions. At the end of the two-week mind-body admission Pearl was mobilising without aids, and her oxycodone was ceased. Her gait, though not yet back to normal, was sufficiently stable to allow her to mobilise independently at her local school.
During the same admission Pearl’s anxiety—in the form of constant ruminations—about her pain and about many other worries and concerns became manifest. It was also noticed that she took hours to get to sleep because of the worry thoughts that went round and round in her mind. She was prescribed melatonin and a small dose of quetiapine to settle her sleep (the melatonin alone was insufficient). She was prescribed an SSRI (fluvoxamine) for the anxiety (titrated up over three weeks), and she was referred to a psychologist in the community for ongoing work pertaining to anxiety. Two months later, when her anxiety was beginning to settle, the melatonin and quetiapine were withdrawn. Two months after that her gabapentin was discontinued.

### 4.9 The management of menstrual-related issues

An important element not captured by the current data is the management of menstrual-related issues. These data were not captured in the current study because menstrual issues were typically identified only during the mind-body admission itself. In such cases, the adolescent was referred to see a gynaecologist after completing the admission.

Sex hormones have differential impacts on the body stress system. Females have a more robust stress system, which enables them to efficiently protect their bodies—and their foetuses—from infection (54, 55). Female sex hormones have a catabolic effect and function to augment the inflammatory/immune response and to stimulate the HPA/sympathetic systems (see Text Box 1 in Kozlowska [2013] for a more detailed summary (56)). By contrast, androgens appear to have mild suppressive effects on the HPA axis and the inflammatory/immune response.

Progesterone, which peaks in the luteal, premenstrual phase of the menstrual cycle, increases minute ventilation—a 25% increase in the luteal phase—and raises the sensitivity of the CO2 respiratory centres (57, 58). Hormone-induced changes in respiration can result in a mild hypocapnia and a shift in the O2 dissociation curve: oxygen molecules bind more tightly to erythrocyte haemoglobin, resulting in decreased tissue O2 availability (Bohr effect) (59). Children with functional seizures are already prone to hyperventilation in response to psychological stress (6, 23). For some children, hyperventilation—along with the physiological changes it yields—is a trigger for their functional seizures (23). What this means for menstruating adolescent girls is that, during the luteal phase of the menstrual cycle, they may be more prone to experiencing functional seizures than in other phases of the cycle. This may be particularly problematic for adolescent girls with irregular periods or very frequent periods. In these cases stabilisation of the cycle using the oral contraceptive pill can be very helpful.

Another body of work has found that sex hormones—including oestrogen, progesterone, and testosterone—are involved in pain processing. Oestrogen acts on the oestrogen receptors on the nerve and immune-inflammatory cells that are part of body’s pain system. Via activation of these receptors, oestrogen appears to play an important role in up-regulating and down-regulating pain. In particular, oestrogen can up-regulate the immune-inflammatory component of the pain system (and stress system more generally) to sustain chronic/complex pain (54). In visceral pain—for example, pain in the abdomen, which was experienced by a tenth of our patients (n = 20; 12.7%)—oestrogen generally has a pro-nociceptive role, intensifying the subjective experience of pain (60, 61). By contrast, progesterone and testosterone appear to have anti-nociceptive effects.

Menstruating adolescent girls experience menstrual pain on a monthly basis, and this pain can contribute to the sense of dis-ease that is experienced as part of the FND illness. Moreover, low iron levels secondary to heavy periods can contribute to comorbid fatigue. In some cases, using tranexamic acid to manage heavy periods can ameliorate the adolescent’s clinical course across the menstrual cycle and can decrease the need for iron supplements for low iron levels. One-tenth of our participants (n = 17; 10.8%) had low iron levels caused by recurrent heavy periods (see **Table 4**).

### 4.10 The challenges of working with subgroup of children with FND comorbid with a neurodevelopmental disorder

In the current study, 12% of children (n = 19; 6 boys, 13 girls)) had previously been diagnosed with ASD. The diagnosis was recorded only in cases where formal testing by a specialist service had yielded the diagnosis. Of the children with ASD, one had an IQ in the borderline range, one in the delayed range, and the rest in the normative range. Twelve other children had IQs in the borderline or delayed range, but without ASD (see **Table 2**).

A recent systematic review found that around 10% of children presenting with FND (functional seizure subtype) had a comorbid ASD diagnosis (62). And a retrospective audit of paediatric cases in the United Kingdom suggests a frequency of 11% (11/97), as well as parent, carer, or clinician concerns pertaining to autistic traits in 33% (32/97) of cases (18). Taken together, these data across three cohorts suggest that children with FND are 3–4 times more likely to have a comorbid diagnosis of ASD than the general population, which the Centers for Disease Control estimates as 1 in 36 (63). Potential explanations pertaining to the association between FND and ASD include shared vulnerability across both disorders pertaining to the following: emotional dysregulation; impaired interoception/interoceptive sensibility (in some cases related to states of high arousal) (51); increased obsessional/neurotic personality traits; and greater cognitive rigidity (making it more difficult for the children to manage adverse life events); and states of high physiological arousal. Interrelated with these factors may be an increased vulnerability to neural network dysregulation (1, 64), which is more likely to occur in children with premorbid conditions that affect brain structure and function. Both epilepsy (comorbid in 20% of patients with FND (65)) and ASD are characterised by some degree of neural network dysregulation (64, 66, 67).

What is evident in clinical practice is that children with neurodevelopmental disorders such as ASD—as well as those with intellectual disability—have vulnerabilities that make the treatment of FND more difficult. In particular, they have significant difficulties in monitoring internal body states (including physiological arousal), identifying and verbalizing emotions, shifting attentional focus, and engaging independently in self-regulation strategies. All of these skills are core elements in the treatment of paediatric FND (6, 15, 33, 68).

In the current study cohort, all children with ASD—and most children with intellectual disability—required a medication adjunct, and their use of medication was at higher levels (greater number of medications) than the rest of the cohort. In this subgroup of children, use of medications to scaffold good sleep and to manage high levels of arousal—so that the child could successfully engage in the functional elements of the program (physiotherapy, group work, attendance at school)—was an essential part of the mind-body intervention for FND.

For examples, see the above vignettes of Elizabetta (section 4.2) and Petter (section 4.4).

### 4.11 The anti-inflammatory properties commonly used psychiatric medications

FND in children is commonly triggered in the context of emotional or physical stress. Common physical trigger events—reported by 41.8% of this sample—include a viral illness, an injury of some sort, or a medical procedure (including vaccination) (69). In this context it is apparent that activation of the immune/inflammatory system by a physical stressor can activate other components of the stress system—including the brain stress system—resulting in the onset of FND symptoms (see Lim and colleagues for more detail)(see **Figure 2** for a visual representation of the overlap between stress-system components).

A number of recent studies suggest that inflammatory processes (24, 70) and oxidative stress (29) are part of the neurobiology of FND. The anti-inflammatory properties of commonly used psychiatric medications may therefore be important in their own right (71, 72). Patel and colleagues (2022) summarise the emerging evidence that SSRIs and serotonin-norepinephrine reuptake inhibitors (SNRIs) modulate inflammation and immune activation, thereby reducing expression of pro-inflammatory cytokines (in particular, IL-6 and TNF-α) (71). SSRIs—for example, escitalopram—may also act on the kynurenine pathway to ameliorate neurotoxicity by increasing the ratio of neuroprotective metabolites (73).

Treatment with atypical anti-psychotics appears to reduce inflammatory cytokines and may sometimes increase the levels of anti-inflammatory cytokines (71). Whether these effects also occur in patients who receive these medications for indications other than psychosis is not known.

### 4.12 Limitations

Medications documented in this study were those that had been formally prescribed by a medical practitioner and that were written up on the hospital medication chart or discharge report. Complementary medicines—non-prescribed over-the-counter vitamins, minerals, herbal teas and remedies, aromatherapy, and homoeopathic preparations—were not systematically recorded and are not reported. The use of PRN paracetamol at time of admission was not systematically recorded and is not reported. Likewise, the use of PRN anti-inflammatory medications on admission is likely to be an under-reported as they were recorded only if written up on the medication chart. That said, most children with complex chronic pain would have used these preparations at some time in their illness journey.

Gynaecology interventions pertaining to oral contraceptive pills, tranexamic acid, and other potential interventions were not captured in this study and are under-reported. The majority of referrals for gynaecology review occurred after completing the Mind-body Program; those interventions were not captured in our study’s database.

Another important limitation is the absence of data pertaining to family beliefs about medication efficacy, both before and after the intervention. Future research on FND should examine the impact of expectations, beliefs, and the placebo/nocebo response on treatment—both process and outcomes. As noted in the introduction, the current study cohorts reflect the more severe end of the illness spectrum: children who were referred to a tertiary care hospital and characterised by high rates of ACEs, high levels of distress, and high rates of psychiatric, functional, and organic medical comorbidity. In this context, the clinical practice described in this article may not have as much relevance to children with FND whose symptoms are transient or who can be treated in an outpatient context.

## 5 Conclusion

The current study reports on the adjunct use of pharmacotherapy in children with FND treated via a rehabilitation approach—the Mind-Body Program—in a tertiary-care hospital setting. Most commonly, medications were used to support good sleep, to down-regulate arousal, to supplement deficiencies, and to treat comorbid psychiatric, functional, and other medical symptoms and disorders. At the heart of it, the mind-body team’s use of medication in the treatment of children with FND is embedded in the broader holistic (biopsychosocial) practice of the Mind-Body Program and in the therapeutic healing ritual that the program represents. Under this framework, medication is never used alone during the mind-body admission itself. Medication use is always combined with concurrent psychotherapy, physiotherapy, school attendance, and family engagement in family work, and the implementation of all of these treatment components as part of the healing ritual. Medication is also framed as a temporary measure for the treatment of FND. The longer-term goal for the child and family is to support them to develop regulation skills and new ways of communicating about stress and distress, thereby decreasing the need for medication and, if necessary, limiting the use of medication to periods of intense pressure or challenge, or to the treatment of comorbid psychiatric, neurodevelopmental, or medical disorders. It is our clinical experience that medication scaffolds the child to allow the child to successfully engage in the interventions that make up the program. It is through this engagement that the child’s agency and mastery—and that of the family—are built. Used on its own, medication is unlikely to result in the therapeutic gains that many children make in the Mind-Body Program (13). When used in conjunction with the other components of the program, medication increases the probability that the child will succeed in the Mind-body Program, thereby embarking on a path to health and well-being.

## Data Availability

The datasets analysed for this study are not publicly available because ethics to place data in a public repository was not obtained from the children and families who participated in this study. Requests to access the datasets should be directed to kkoz6421@uni.sydney.edu.au.

## References

[1] Rai S Foster S Griffiths KR Breukelaar IA Kozlowska K Korgaonkar MS. Altered resting-state neural networks in children and adolescents with functional neurological disorder. NeuroImage Clin. (2022) 35:103110. doi: 10.1016/j.nicl.2022.103110, PMID: 36002964 PMC9421459

[2] Vassilopoulos A Mohammad S Dure L Kozlowska K Fobian AD. Treatment approaches for functional neurological disorders in children. Curr Treat Options Neurol. (2022) 24:77–97. doi: 10.1007/s11940-022-00708-5, PMID: 35370394 PMC8958484

[3] Kozlowska K Scher S. Recent advances in understanding the neurobiology of pediatric functional neurological disorder. Expert Rev Neurother. (2024) 24(5):497–516. doi: 10.1080/14737175.2024.2333390, PMID: 38591353

[4] Kozlowska K Chudleigh C Savage B Hawkes C Scher S Nunn KP. Evidence-based mind-body interventions for children and adolescents with functional neurological disorder. Harv Rev Psychiatry. (2023) 31:60–82. doi: 10.1097/HRP.0000000000000358, PMID: 36884038 PMC9997641

[5] Lazarus AA. Multimodal behavior therapy. New York: Springer Pub. Co (1976). p. 241.

[6] Kozlowska K Scher S Helgeland H. Functional somatic symptoms in children and adolescents: A stress-system approach to assessment and treatment. London: Palgrave Macmillan (2020).

[7] Keynejad RC Frodl T Kanaan R Pariante C Reuber M Nicholson TR. Stress and functional neurological disorders: mechanistic insights. J Neurol Neurosurg Psychiatry. (2019) 90:813–21. doi: 10.1136/jnnp-2018-318297, PMID: 30409887

[8] Weber S Buhler J Vanini G Loukas S Bruckmaier R Aybek S. Identification of biopsychological trait markers in functional neurological disorders. Brain. (2023) 146:2627–41. doi: 10.1093/brain/awac442, PMID: 36417451 PMC10232283

[9] Sharma AA Szaflarski JP. Neuroinflammation as a pathophysiological factor in the development and maintenance of functional seizures: A hypothesis. Epilepsy Behav Rep. (2021) 16:100496. doi: 10.1016/j.ebr.2021.100496, PMID: 34917920 PMC8645839

[10] Agorastos A Pervanidou P Chrousos GP Baker DG. Developmental trajectories of early life stress and trauma: A narrative review on neurobiological aspects beyond stress system dysregulation. Front Psychiatry. (2019) 10:118. doi: 10.3389/fpsyt.2019.00118, PMID: 30914979 PMC6421311

[11] Chung J Mukerji S Kozlowska K. Cortisol and alpha-amylase awakening response in children and adolescents with functional neurological (conversion) disorder. Aust N Z J Psychiatry. (2023) 57(1):115–29. doi: 10.1177/00048674221082520, PMID: 35297291

[12] McCraty R Childre D. Coherence: bridging personal, social, and global health. Altern Ther Health Med. (2010) 16:10–24., PMID: 20653292

[13] Kozlowska K Gray N Scher S Savage B. Psychologically informed physiotherapy as part of a multidisciplinary rehabilitation program for children and adolescents with functional neurological disorder: Physical and mental health outcomes. J Paediatr Child Health. (2021) 57:73–9. doi: 10.1111/jpc.15122, PMID: 32861224

[14] Kozlowska K Schollar-Root O Savage B Hawkes C Chudleigh C Raghunandan J. Illness-promoting psychological processes in children and adolescents with functional neurological disorder. Children (Basel). (2023) 10:1724. doi: 10.3390/children10111724, PMID: 38002815 PMC10670544

[15] Fobian AD Long DM Szaflarski JP. Retraining and control therapy for pediatric psychogenic non-epileptic seizures. Ann Clin Transl Neurol. (2020) 7(8):1410–9. doi: 10.1002/acn3.51138, PMID: 32748572 PMC7448150

[16] Gianaros PJ Wager TD. Brain-body pathways linking psychological stress and physical health. Curr Dir Psychol Sci. (2015) 24:313–21. doi: 10.1177/0963721415581476, PMID: 26279608 PMC4535428

[17] Prince E Kim E Wall C Gisin E Goodwin M Schoen Simmons E. The relationship between autism symptoms and arousal level in toddlers with autism spectrum disorder, as measured by electrodermal activity. Autism. (2017) 21(4):504–8. doi: 10.1177/1362361316648816, PMID: 27289132 PMC5812779

[18] Simpson A Tallur KK Chin RFM Yong K Stone J. Autism spectrum disorder in children and young people with FND. J Psychosom Res. (2024) 182:111681. doi: 10.1016/j.jpsychores.2024.111681, PMID: 38692183

[19] AmericanPsychiatric A. Diagnostic and statistical manual of mental disorders: DSM-IV-TR. 4th ed. Washington, DC: American Psychiatric Association (2000). p. 943.

[20] AmericanPsychiatricAssociation. Diagnostic and statistical manual of mental disorders: DSM-5. 4th ed. Arlington, VA: American Psychiatric Association (2013).

[21] Kozlowska K Palmer DM Brown KJ McLean L Scher S Gevirtz R. Reduction of autonomic regulation in children and adolescents with conversion disorders. Psychosom Med. (2015) 77:356–70. doi: 10.1097/PSY.0000000000000184, PMID: 25954919

[22] Wells R Spurrier AJ Linz D Gallagher C Mahajan R Sanders P. Postural tachycardia syndrome: current perspectives. Vasc Health Risk Manage. (2018) 14:1–11. doi: 10.2147/VHRM.S127393, PMID: 29343965 PMC5749569

[23] Kozlowska K Rampersad R Cruz C Shah U Chudleigh C Soe S. The respiratory control of carbon dioxide in children and adolescents referred for treatment of psychogenic non-epileptic seizures. Eur Child Adolesc Psychiatry. (2017) 26:1207–17. doi: 10.1007/s00787-017-0976-0, PMID: 28341888 PMC5610228

[24] Kozlowska K Chung J Cruickshank B McLean L Scher S Dale RC. Blood CRP levels are elevated in children and adolescents with functional neurological symptom disorder. Eur Child Adolesc Psychiatry. (2019) 28:491–504. doi: 10.1007/s00787-018-1212-2, PMID: 30143887

[25] Radmanesh M Jalili M Kozlowska K. Activation of functional brain networks in children and adolescents with psychogenic non-epileptic seizures. Front Hum Neurosci. (2020) 14:339. doi: 10.3389/fnhum.2020.00339, PMID: 33192376 PMC7477327

[26] Kozlowska K Melkonian D Spooner CJ Scher S Meares R. Cortical arousal in children and adolescents with functional neurological symptoms during the auditory oddball task. NeuroImage Clin. (2017) 13:228–36. doi: 10.1016/j.nicl.2016.10.016, PMID: 28003962 PMC5157791

[27] Kozlowska K Griffiths KR Foster SL Linton J Williams LM Korgaonkar MS. Grey matter abnormalities in children and adolescents with functional neurological symptom disorder. NeuroImage Clin. (2017) 15:306–14. doi: 10.1016/j.nicl.2017.04.028, PMID: 28560155 PMC5440356

[28] Charney M Foster S Shukla V Zhao W Jiang SH Kozlowska K. Neurometabolic alterations in children and adolescents with functional neurological disorder. NeuroImage: Clin. (2024) 41:103557. doi: 10.1016/j.nicl.2023.103557, PMID: 38219534 PMC10825645

[29] Lan Z Foster S Charney M van Grinsven M Breedlove K Kozlowska K. Neurometabolic network (NMetNet) for functional neurological disorder in children and adolescents. NeuroImage Clin. (2025) 46:103767. doi: 10.1016/j.nicl.2025.103767, PMID: 40187194 PMC12002944

[30] Fleming S Thompson M Stevens R Heneghan C Pluddemann A Maconochie I. Normal ranges of heart rate and respiratory rate in children from birth to 18 years of age: a systematic review of observational studies. Lancet. (2011) 377:1011–8. doi: 10.1016/S0140-6736(10)62226-X, PMID: 21411136 PMC3789232

[31] Hyams JS Di Lorenzo C Saps M Shulman RJ Staiano A van Tilburg M. Functional disorders: children and adolescents. Gastroenterology. (2016). doi: 10.1053/j.gastro.2016.02.015, PMID: 27144632

[32] Schondorf R Low PA. Idiopathic postural orthostatic tachycardia syndrome: an attenuated form of acute pandysautonomia? Gastroenterology. (2016) 15:S0016-5085(16)00181-5. doi: 10.1053/j.gastro.2016.02.015, PMID: 8423877

[33] Savage B Chudleigh C Hawkes C Scher S Kozlowska K. Treatment of functional seizures in children and adolescents: A mind-body manual for health professionals. Samford Valley, Queensland: Australian Academic Press (2022).

[34] Stahl SM Strawn JR. Stahl’s Essential Psychopharmacology Prescriber’s Guide Children and Adolescents 2nd ed 2024 Stephen M Stahl and Jeffrey Strawn Cambridge University press. 2nd ed. Cambridge, UK: Cambridge University Press (2024).

[35] Helgeland H Savage B Kozlowska K. Hypnosis in the treatment of functional somatic symptoms in children and adolescents. In: Linden JH De Benedittis G Sugarman LI Varga K, editors. The routledge international handbook of clinical hypnosis. New York: Routledge Taylor & Francis (2024). p. 515–36.

[36] Dodd S Dean OM Vian J Berk M. A review of the theoretical and biological understanding of the nocebo and placebo phenomena. Clin Ther. (2017) 39:469–76. doi: 10.1016/j.clinthera.2017.01.010, PMID: 28161116

[37] Cirelli C. Sleep and synaptic changes. Curr Opin Neurobiol. (2013) 23:841–6. doi: 10.1016/j.conb.2013.04.001, PMID: 23623392 PMC4552336

[38] Tononi G Cirelli C. Sleep and the price of plasticity: from synaptic and cellular homeostasis to memory consolidation and integration. Neuron. (2014) 81:12–34. doi: 10.1016/j.neuron.2013.12.025, PMID: 24411729 PMC3921176

[39] NICE. Depression in children and young people: identification and management. In: National Institute for Health and Care Excellence (NICE) guideline [NG134]. London, UK: National Institute for Health and Care Excellence (NICE). (2019). Available at: https://www.nice.org.uk/guidance/ng134/chapter/Recommendationssteps-4-and-5-managing-moderate-to-severe-depression. (Accessed January 12, 2025) 31577402

[40] Kopcalic K Arcaro J Pinto A Ali S Barbui C Curatoli C. Antidepressants versus placebo for generalised anxiety disorder (GAD). Cochrane Database Syst Rev. (2025) 1(1):CD012942. doi: 10.1002/14651858, PMID: 39880377 PMC11779548

[41] Kim Y-N Gray N Jones A Scher S Kozlowska K. The role of physiotherapy in the management of functional neurological disorder in children and adolescents. Semin Pediatr Neurol. (2022) 41:100947. doi: 10.1016/j.spen.2021.100947, PMID: 35450664

[42] Hebb DO. The organization of behavior: a neuropsychological theory. Santo Domingo JM, editor. New York: Wiley (1949).

[43] Wu YK Zenke F. Nonlinear transient amplification in recurrent neural networks with short-term plasticity. eLife. (2021) 10:e71263. doi: 10.7554/eLife.71263, PMID: 34895468 PMC8820736

[44] Neuroscience News. How neurons that wire together fire together (2021). Available online at: https://neurosciencenews.com/wire-fire-neurons-19835/ (Accessed December 6, 2024).

[45] Emslie GJ Heiligenstein JH Wagner KD Hoog SL Ernest DE Brown E. Fluoxetine for acute treatment of depression in children and adolescents: A placebo-controlled, randomized clinical trial. J Am Acad Child Adolesc Psychiatry. (2002) 41:1205–15. doi: 10.1097/00004583-200210000-00010, PMID: 12364842

[46] Paredes-Echeverri S Maggio J Begue I Pick S Nicholson TR Perez DL. Autonomic, endocrine, and inflammation profiles in functional neurological disorder: A systematic review and meta-analysis. J Neuropsychiatry Clin Neurosci. (2022) 34:30–43. doi: 10.1176/appi.neuropsych.21010025, PMID: 34711069 PMC8813876

[47] Wilson LD Leath TC Patel MB. Management of paroxysmal sympathetic hyperactivity after traumatic brain injury. In: Heidenreich K, editor. New therapeutics for traumatic brain injury: prevention of secondary brain damage and enhancement of repair and regeneration. Academic Press, Amsterdam, [Netherlands] (2017). p. 145–58.

[48] Stahl SM. Essential psychopharmacology neuroscientific basis and practical applications. New York: Cambridge University Press (2021).

[49] Burridge N Symons K. Australian don’t rush to crush handbook. 3rd ed. Collingwood Collingwood, Victoria: Society of Hospital Pharmacists of Australia (2018).

[50] Strawn J Keeshin B Cohen JA. Posttraumatic stress disorder in children and adolescents: Treatment overview UpToDate 2024 Last updated 6th September 2023 (2023). NSW CIAP.

[51] Tyrer A Ehmsen J Hoogervorst K Nikolova N Pando-Naude V Steenkjaer C. Peripheral beta-blockade differentially enhances cardiac and respiratory interoception. bioRxiv. (2025), 2025.02.28.640776. doi: 10.1101/2025.02.28.640776

[52] Vachon-Presseau E Centeno MV Ren W Berger SE Tetreault P Ghantous M. The emotional brain as a predictor and amplifier of chronic pain. J Dent Res. (2016) 95:605–12. doi: 10.1177/0022034516638027, PMID: 26965423 PMC4924545

[53] Hazell P Mirzaie M Hazell P. Tricyclic drugs for depression in children and adolescents. Cochrane Database Syst Rev. (2013) 2013:CD002317–CD. doi: 10.1002/14651858.CD002317.pub2, PMID: 23780719 PMC7093893

[54] Chrousos GP. Stress and sex versus immunity and inflammation. Sci Signal. (2010) 3:pe36. doi: 10.1126/scisignal.3143pe36, PMID: 20940425

[55] Whitacre CC Reingold SC O’Looney PA Blankenhorn E Brinley F Collier E. A gender gap in autoimmunity: task force on gender, multiple sclerosis and autoimmunity. Sci (American Assoc Advancement Science). (1999) 283:1277–8. doi: 10.1126/science.283.5406.1277, PMID: 10084932

[56] Kozlowska K. Stress, distress, and bodytalk: co-constructing formulations with patients who present with somatic symptoms. Harv Rev Psychiatry. (2013) 21:314–33. doi: 10.1097/HRP.0000000000000008, PMID: 24201822

[57] Damas-Mora J Davies L Taylor W Jenner FA. Menstrual respiratory changes and symptoms. Br J Psychiatry. (1980) 136:492–7. doi: 10.1192/bjp.136.5.492, PMID: 6770935

[58] Lyons HA. Respiratory effects of gonadal hormones. In: Salhanick HA Kipnis DM Van de Wiele RL, editors. Metabolic effects of gonadal hormones and contraceptive steroids. Plenum Press, New York (1969). p. 394–402.

[59] Bohr C Hasselbalch K Krogh A. Über einen in biologischer Beziehung wichtigen Einfluss, den die Kohlensäurespannung des Blutes auf dessen Sauerstoffbindung übt [Concerning a Biologically Important Relationship -The Influence of the Carbon Dioxide Content of Blood on its Oxygen Binding. Skandin Arch Physiol. (1904) 16:401–12.

[60] Sun LH Zhang WX Xu Q Wu H Jiao CC Chen XZ. Estrogen modulation of visceral pain. J Zhejiang Univ Sci B. (2019) 20:628–36. doi: 10.1631/jzus.B1800582, PMID: 31273960 PMC6656561

[61] Traub RJ Ji Y. Sex differences and hormonal modulation of deep tissue pain. Front Neuroendocrinol. (2013) 34:350–66. doi: 10.1016/j.yfrne.2013.07.002, PMID: 23872333 PMC3830473

[62] Vickers ML Menhinnitt RS Choi YK Malacova E Eriksson L Churchill AW. Comorbidity rates of autism spectrum disorder and functional neurological disorders: A systematic review, meta-analysis of proportions and qualitative synthesis. Autism: Int J Res Pract. (2025) 29(2):344–54. doi: 10.1177/13623613241272958, PMID: 39152614

[63] Maenner MJ Warren Z Williams AR Amoakohene E Bakian AV Bilder DA. Prevalence and characteristics of autism spectrum disorder among children aged 8 years — Autism and developmental disabilities monitoring network, 11 sites, United States, 2020. MMWR Surveillance Summaries. (2023) 72:1–14. doi: 10.15585/mmwr.ss7202a1, PMID: 36952288 PMC10042614

[64] Shan X Wang P Yin Q Li Y Wang X Feng Y. Atypical dynamic neural configuration in autism spectrum disorder and its relationship to gene expression profiles. Eur Child Adolesc Psychiatry. (2025) 34:169–79. doi: 10.1007/s00787-024-02476-w, PMID: 38861168

[65] Carle-Toulemonde G Goutte J Do-Quang-Cantagrel N Mouchabac S Joly C Garcin B. Overall comorbidities in functional neurological disorder: A narrative review. Encephale. (2023) 49:S24–32. doi: 10.1016/j.encep.2023.06.004, PMID: 37414721

[66] Chen Z Wang X Zhang S Han F. Neuroplasticity of children in autism spectrum disorder. Front Psychiatry. (2024) 15:1362288–. doi: 10.3389/fpsyt.2024.1362288, PMID: 38726381 PMC11079289

[67] San-Juan D Rodríguez-Méndez D. Epilepsy as a disease affecting neural networks: A neurophysiological perspective. Neurologia (Barcelona Spain). (2023) 38(2):114–23. doi: 10.1016/j.nrleng.2020.06.016, PMID: 36396092

[68] Sawchuk T Buchhalter J Senft B. Psychogenic nonepileptic seizures in children-Prospective validation of a clinical care pathway & risk factors for treatment outcome. Epilepsy Behav. (2020) 105:106971. doi: 10.1016/j.yebeh.2020.106971, PMID: 32126506

[69] Lim N Wood N Prasad A Waters K Singh-Grewal D Dale RC. COVID-19 vaccination in young people with functional neurological disorder: A case-control study. Vaccines (Basel). (2022) 10(12):2031. doi: 10.3390/vaccines10122031, PMID: 36560442 PMC9782633

[70] Gledhill JM Brand EJ Pollard JR St Clair RD Wallach TM Crino PB. Association of epileptic and nonepileptic seizures and changes in circulating plasma proteins linked to neuroinflammation. Neurology. (2021) 96:e1443–e52. doi: 10.1212/WNL.0000000000011552, PMID: 33495377 PMC8055314

[71] Patel S Keating BA Dale RC. Anti-inflammatory properties of commonly used psychiatric drugs. Front Neurosci. (2022) 16:1039379. doi: 10.3389/fnins.2022.1039379, PMID: 36704001 PMC9871790

[72] Hannestad J DellaGioia N Bloch M. The effect of antidepressant medication treatment on serum levels of inflammatory cytokines: A meta-analysis. Neuropsychopharmacology. (2011) 36:2452–9. doi: 10.1038/npp.2011.132, PMID: 21796103 PMC3194072

[73] Halaris A Myint AM Savant V Meresh E Lim E Guillemin G. Does escitalopram reduce neurotoxicity in major depression? J Psychiatr Res. (2015) 66-67:118–26. doi: 10.1016/j.jpsychires.2015.04.026, PMID: 26009299

[74] Lovibond SH Lovibond PF. Manual for the depression anxiety stress scale. Sydney: The Psychological Foundation of Australia, Inc (1995).

[75] Patrick J Dyck M Bramston P. Depression anxiety stress scale: is it valid for children and adolescents? J Clin Psychol. (2010) 66:996–1007. doi: 10.1002/jclp.20696, PMID: 20694962

[76] Cohen RA Hitsman BL Paul RH McCaffery J Stroud L Sweet L. Early life stress and adult emotional experience: an international perspective. Int J Psychiatry Med. (2006) 36:35–52. doi: 10.2190/5R62-9PQY-0NEL-TLPA, PMID: 16927577

